# Bioinformatics-driven insights: rapamycin-mediated CaMK2D inhibition alleviates intestinal ischemia-reperfusion injury

**DOI:** 10.3389/fimmu.2025.1684853

**Published:** 2026-05-01

**Authors:** Ruxiang Sheng, Yanqiu Liang, Huihong Zhang, Yonghe Lai, Haiyun Hong, Dingbang Huang, Dezhao Liu

**Affiliations:** Department of Anesthesiology, Fifth Affiliated Hospital of Sun Yat-Sen University, Zhuhai, Guangdong, China

**Keywords:** calcium/calmodulin dependent-protein kinase IIδ, genomics, inflammation, intestinal ischemia-reperfusion injury, rapamycin

## Abstract

**Introduction:**

Intestinal ischemia-reperfusion (I/R) injury, a common and severe clinical condition with high morbidity and mortality, burdens healthcare systems. Our previous investigations established that a nano-delivery system enabled targeted rapamycin delivery to intestinal I/R injury sites with therapeutic efficacy. While calcium/calmodulin-dependent protein kinase IIδ (CaMK2D) has been implicated in myocardial injury and tumorigenesis, its role in intestinal I/R pathophysiology remains unexplored. This study investigates the therapeutic mechanisms of rapamycin in intestinal I/R injury by modulation of CaMK2D signaling.

**Methods:**

An oxygen-glucose deprivation/reperfusion (OGD/R) model in Caco-2 human colorectal cancer cells and a murine intestinal I/R model were established. Small interfering RNA (siRNA) and hesperadin (HES) were used to inhibit CaMK2D expression. Transcriptomic profiling was performed via RNA sequencing (RNA-Seq) with subsequent bioinformatic analysis including differential gene expression, MCODE-based protein interaction network clustering, and RAPA-CaMK2D molecular docking studies. Cellular assays included qRT - PCR, western blotting (WB), Fluo-3 calcium flux analysis, flow cytometry, and Enzyme-linked immunosorbent assay (ELISA). In animal experiments, HE staining, immunohistochemistry, TUNEL assay, WB, and ELISA were employed.

**Results:**

Both cellular and murine models demonstrated a significant upregulation of CaMK2D phosphorylation with intestinal epithelial apoptosis, barrier dysfunction, and enhanced inflammatory response during I/R. CaMK2D knockdown using siRNA attenuated these pathological manifestations, vice versa. Bioinformatic analysis revealed a CaMK2D-dominated regulatory module (ranked fifth) enriched in calcium-mediated signaling pathways. Mechanistically, I/R induced CaMK2D activation exacerbated inflammatory cascades, epithelial apoptosis, and tight junction disruption. Rapamycin treatment (1.5 mg/kg, i.p.) ameliorated these effects by decreasing CaMK2D expression and phosphorylation (WB, *P* < 0.01), pro-inflammatory cytokine levels (ELISA, *P* < 0.01), while preserving intestinal integrity as evidenced by histological analysis (IHC, *P* < 0.05).

**Discussion:**

Our findings establish CaMK2D hyperactivation as a key to intestinal I/R injury. The therapeutic potential of rapamycin derived from its ability to suppress CaMK2D signaling axis, providing a novel pharmacological strategy for intestinal I/R management.

## 1 Introduction

Intestinal I/R injury constitutes a critical clinical challenge following trauma, infection, mesenteric thrombosis, transplantation, and sepsis (1, 2). Pathological hallmarks include a large amount of apoptosis and disruption of intestinal barrier function, causing gut microbiota and endotoxins breaching the intestinal barrier and translocating, which in turn affects circulatory system and distant organs. This triggers the systemic inflammatory response syndrome (SIRS), multiple organ dysfunction syndrome (MODS), and even results in death (3). The occurrence and development of such serious consequences are closely related to the damage of intestinal structure and function. Research has verified that the integrity of intestinal epithelial cells (IECs) is crucial for maintaining the intestinal barrier function, and the extent of IEC damage is closely correlated with the clinical prognosis (4). The current treatment strategies are grounded in the restoration of blood flow, encompassing surgical revascularization procedures and pharmacological interventions. Nevertheless, owing to the intricate mechanisms underlying I/R injury, which entail multiple signaling pathways such as abnormal mitochondrial dynamics, apoptosis, and pyroptosis, the mortality rate associated with this condition reaches as high as 50% (5). Consequently, gaining a profound understanding of the mechanisms underlying intestinal I/R injury is of paramount significance.

CaMK2, a multifunctional serine/threonine kinase, demonstrates widespread tissue distribution and regulatory roles in cellular calcium homeostasis, apoptotic pathways, and a transcriptional regulation (6). During the I/R process, intracellular calcium overload and the burst of reactive oxygen species (ROS) frequently induce the activation of CaMKII. In the context of myocardial I/R injury or heart failure, receptor-interacting protein kinase 3 (RIPK3) mediates the necroptosis of cardiomyocytes via the activation of CaMK2D (7). Simultaneously, the overexpression of CaMK2D results in the activation of nuclear factor kappa-light-chain-enhancer of activated B cells (NF-κB), and the ensuing inflammatory response inflicts persistent damage upon cardiomyocytes (8). Notably, in the hypoxia/reoxygenation (H/R) model of cardiomyocytes, the inhibition of CaMK2D can remarkably enhance the autophagic flux and effectively mitigate the autophagic dysfunction triggered by I/R (9). Whiles these cardiac findings establish CaMK2D as a therapeutic target, its functional role in intestinal I/R injury remains underexplored. Current literature predominantly focuses on cardiac applications, leaving a critical knowledge gap regarding intestinal epithelial responses to ischemic stress.

Our previous research developed a ROS-responsive nanoparticle-based drug delivery system targeting intestinal I/R injury, engineered to selectively release rapamycin (RAPA) at ischemic loci (10). RAPA, a member of the macrolide-class immunosuppressants, was secreted by Streptomyces hygroscopicus and was alternatively named sirolimus. Initially employed as an antifungal agent, it has progressively found applications in multiple fields, including organ transplantation, anti-inflammatory therapy, anti-aging research, and the treatment of cardiovascular diseases. In the course of I/R, owing to mechanisms such as energy exhaustion, calcium overload, oxidative stress, and the outburst of ROS, IECs experience necroptosis and apoptosis. Therefore, the barrier function of the tight junctions in the intestinal epithelium is compromised. RAPA exerts an impact on the transcription of transcription factor EB (TFEB), thereby influencing the expression of phenazine biosynthesis-like domain protein (PBLD), which is specifically deficient in the intestinal epithelium. Leveraging this property, RAPA upregulates the expression level of tight-junction proteins (11). Moreover, a study on lymphoproliferative patients with loss-of-function mutations in stromal interaction molecule (STIM) has revealed that the activation and proliferation capabilities of T cells are enhanced following the administration of RAPA (12). This finding implies that RAPA may possess potential mechanisms for regulating calcium homeostasis, which necessitates further exploration.

**Scheme 1:**
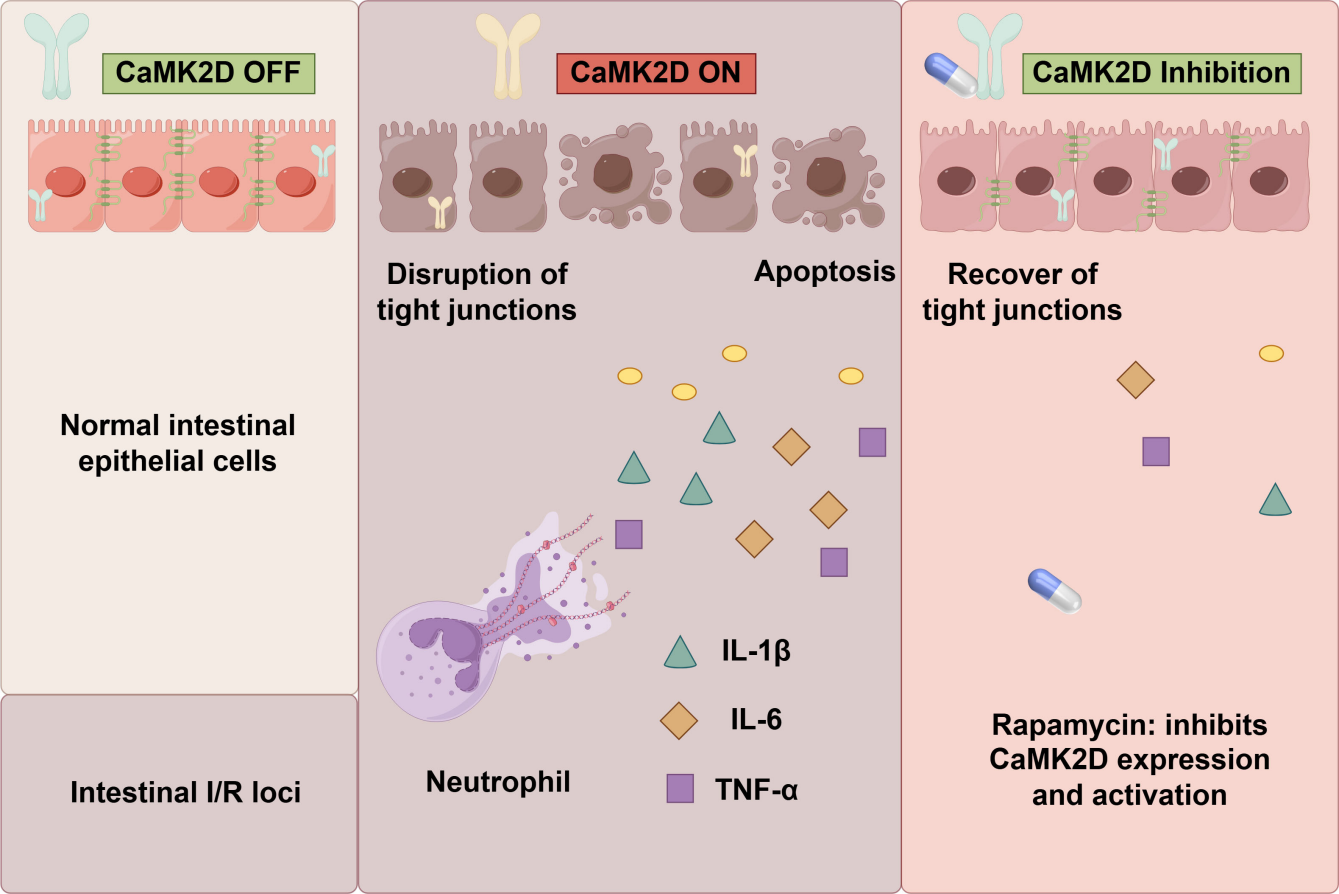
Schematic illustration of rapamycin therapy for intestinal ischemia-reperfusion injury through CaMK2D modulation. Both *in vitro* and *in vivo* experiments indicated that elevated CaMK2D expression was linked to injury severity. Inhibition of CaMK2D significantly mitigated the injury. Rapamycin, by downregulating CaMK2D, effectively exerted a protective effect. Evidently, CaMK2D is a critical determinant in intestinal I/R injury, and rapamycin emerges as a promising therapeutic option for managing this condition.

Herein, we postulated that RAPA might mitigate intestinal I/R injury through the regulation of CaMK2D. The current systematically explored the mechanisms underlying the impact of RAPA on CaMK2D and the intestinal barrier function. These investigations were carried out using an OGD/R model in Caco-2 cells and an *in vivo* animal intestinal I/R model (**Scheme 1**). The aim of this research was to provide novel mechanistic insights and potential therapeutic modalities for the management of intestinal I/R injury.

## 2 Materials and methods

### 2.1 Animals and materials

CaMK2D inhibitor HES was purchased from Beyotime Co., Ltd (Shanghai, China). Methanol and ethanol were obtained from Machlin Biochemistry (Shanghai, China). RAPA was obtained from Coolaber Science & Technology Co., Ltd (Beijing, China). Calcein/PI cell viability assay kit was procured from Beyotime Co., Ltd (Shanghai, China). The ELISA kits for IL-1β, IL-6, and TNF-α were obtained from Bolaz Biotechnology Co., Ltd. (Nanjing, China). The following antibodies were procured from Affinity Biosciences (Melbourne, AUS): a rabbit polyclonal antibody to CaMK2, a rabbit polyclonal antibody to CaMK2B/C/D (Thr287), a rabbit antibody to β-actin, and a goat monoclonal antibody to rabbit IgG. The following antibodies were purchased from Servicebio (Wuhan, China): a rabbit antibody to ZO-1 tight junction protein, a rabbit antibody to OCCLUDIN tight junction protein.

Vascular clips were purchased from Kerong Biotechnology Co., Ltd. (Guangzhou, China).

Cell lines: Caco-2 human colorectal cancer cells were purchased from Servicebio Biology Co., Ltd. (Wuhan, China). Caco-2 cells were cultured in DMEM medium containing 100 mg mL^-1^ streptomycin, 10% fetal bovine serum (FBS), and 100 U mL^-1^ penicillin. The cells were raised at a temperature of 37°C in a controlled environment with added moisture and 5% CO_2_.

Animals: Male C57BL/6 mice with an age of 8 weeks, were purchased from Ruige Biotech Co., Ltd. (Guangzhou, China). All interventions and animal care protocols were carried out in strict accordance with the Animal Surgery Guidelines and Policies for provided by the Animal-related Ethics Panel of the Fifth Affiliated Hospital, Sun Yat-sen University (No. 00591) (Zhuhai, China). The mice were housed in a controlled environment maintained at a temperature range of 24-25 °C, and a humidity level of 55%. A 12-hour light/12-hour dark cycle was implemented, and the mice had ad libitum access to food and water.

### 2.2 Caco-2 cell OGD/R model

Caco-2 cells were plated in six-well plates at a density of 2×10^5^ cells per well. The cultivation continued until the cells achieved a confluency ranging from 70% to 80%. After that, the cells were washed with PBS, and the medium was replaced with PBS. The cells were then incubated in a hypoxia incubator (Binder) for 12 hours. Following this, the medium was changed to DMEM universal medium and the cells were further incubated for two hours (13). After the aforementioned treatments, the cells were grouped as follows: Control group: Cells were cultured under normal conditions without OGD/R treatment. OGD/R group: Cells underwent pure OGD/R treatment without intervention. RAPA group: Cells were subjected to OGD/R treatment and co-incubated with 0.94 μg mL^-1^ RAPA (based on our previous research (10)) for 2 h during the reoxygenation phase. oe+RAPA group: Cells were transfected with 2.0 μg/well (6-well plate) t-CaMK2D overexpression plasmid (optimal concentration confirmed by dose-response assay), cultured for 48 h, then subjected to OGD/R treatment. During the reoxygenation phase, cells were co-incubated with 0.94 μg mL^-^¹ RAPA for 2 h.

### 2.3 Murine intestinal I/R injury model

The mouse intestinal I/R model was established as follows: The mice were anesthetized via intraperitoneal injection of 3% pentobarbital sodium. The superior mesenteric artery (SMA) was carefully exposed and securely ligated at its distal end using a clamp. After occluding the blood flow to the SMA for 1 hour, the vascular clip was removed to permit reperfusion for an additional 24 hours. In the control group, the vascular clip was removed immediately upon accessing the SMA (14). Mice were randomly separated into four groups, each consisting of three mice. Experimental groups received the following treatments: Sham (0.4 mL saline, i.p.), I/R (0.4 mL saline, i.p.), I/R+HES (5 μg kg^-1^, 0.4 mL, i.p.) (15), I/R+RAPA (1.5 mg kg^-1^, 0.4 mL, i.p.).

### 2.4 Cell viability

Cell viability was determined using a live/dead staining assay, specifically with a Calcein-AM/Propidium Iodide (PI) staining kit. Calcein-AM can be taken up by live cells and hydrolyzed by intracellular esterases to produce green fluorescence, while PI can penetrate the membranes of dead cells and bind to nucleic acids, emitting red fluorescence. Six-well plates were incubated with 2×10^5^ Caco-2 cells/well and cultivated until they reached a confluence of 70-80%. Subsequently, the cells were rinsed using PBS. Then, a staining working solution was prepared according to the kit instructions. 500 μL of the staining working solution was added to each well, and the cells were incubated at 37 °C in the dark for 30 minutes. Thereafter, the cells were rinsed using PBS once more to remove the excess staining solution. The stained cells were then observed under a fluorescence microscope.

### 2.5 Histology, IHC and immunofluorescence

Fresh terminal ileal tissue samples were immersed in 4% paraformaldehyde for histological examination and then embedded in paraffin. Hematoxylin and eosin (H&E) staining was carried out on the intestinal paraffin sections following standard procedures. Two pathologists utilized the Chiu’s scoring system to assess the damage of the intestinal mucosa (16).

For the detection of tight junction proteins OCCLUDIN and ZO-1, after the intestinal paraffin sections were prepared as described above, immunohistochemical staining was performed. First, the sections were deparaffinized and rehydrated. Then, antigen retrieval was carried out using a suitable method, such as heating in a citrate buffer solution. Endogenous peroxidase activity was blocked with hydrogen peroxide. Next, the sections were incubated with primary antibodies against OCCLUDIN and ZO-1 respectively at an appropriate dilution (e.g., OCCLUDIN antibody at 1:200 and ZO-1 antibody at 1:150) overnight at 4 °C. After washing with PBS, the sections were incubated with secondary antibodies conjugated with horseradish peroxidase for a specific period, usually around 30 minutes at room temperature. Finally, the color was developed using a DAB (3,3’-Diaminobenzidine) kit, and the sections were counterstained with hematoxylin. The expression levels of OCCLUDIN and ZO-1 were evaluated by observing the intensity and distribution of the staining under a light microscope. The semi-quantitative analysis could be carried out by calculating the integrated optical density (IOD) of the stained areas using image analysis software.

The intestinal sections were fixed with a 4% paraformaldehyde solution and embedded in paraffin. Terminal deoxynucleotidyltransferase (TdT)-mediated dUDP-biotin nick end labeling (TUNEL) was used to identify apoptotic cells. The apoptotic level was determined by calculating the apoptotic index, which is the proportion of TUNEL-positive cells to the total number of cells.cells.

### 2.6 Isolation of IECs

A modified method was employed to isolate IECs (17). First, the intestinal tissues of mice from previous steps were washed with cold PBS. Then, they were longitudinally incised and cut into 2–3 cm pieces. These pieces were co-incubated with 30 mM EDTA-PBS on ice for 30 min, with vigorous shaking every 10 min. The isolated crypts were rinsed with cold PBS and dissociated with TrypLE Express (Invitrogen) at room temperature for 10 min. The resulting cell suspension was filtered through a 70-μm cell strainer. For flow cytometry sorting, the cell suspension was labeled with a fluorescent-antibody cocktail, including APC-CD45 (Cat. No.17-0451-83; Invitrogen), FITC-CD31 (Cat. No.102406; BioLegend), PE/Cy7-TER-119 (Cat. No.116222; BioLegend), and PE-EpCAM (Cat. No.12-5791-83; Invitrogen). To exclude dead cells, the LIVE/DEAD Fixable Violet Dead Cell Stain (Invitrogen) was used. CD45^-^ CD31^-^ TER-119^-^ EpCAM^+^ endothelial cells were sorted through a SONY SH800S or CytoFLEX SRT Cell Sorter.

### 2.7 Measurement of cytokines

Samples were obtained from serum or cell culture supernatant. The levels of TNF-α, IL-6, and IL-1β in the specimens were measured using an ELISA kit according to the provided instructions.

### 2.8 RNA sequencing and data analysis

RNA-seq was carried out on Caco-2 cells either incubated with or without RAPA. Total RNA from each sample was extracted using TRIzol reagent (catalog number 15596026, Invitrogen, USA). RNA libraries were sequenced on an Illumina sequencing platform provided by Gene Paiseek Biotechnology Co., Ltd. in Shanghai, China. DEGs were clustered through the Molecular Complex Detection (MCODE) algorithm. Moreover, KEGG and GO annotations were employed to determine the relevant pathways of the DEGs.

### 2.9 Molecular docking

The search criteria on UniProt (https://www.uniprot.org/) were restricted to Homo sapiens, and the accession number for CaMK2D was D6R938. Subsequently, the protein number D6R938 was inputted into the RCSB PDB (https://www.rcsb.org/) to retrieve and download specific structure. With the assistance of PyMOL 3.0.3 software, the crystal water molecules within the protein were eliminated. The 2D structure file of RAPA (SID 5284616) obtained from PubChem (https://pubchem.ncbi.nlm.nih.gov/) was converted into a 3D format and then energy-minimized to the lowest energy state using ChenBio3D ultra 14.0 software. AutoDockTools 1.5.7 software was employed to define the binding site, calculate the geometric center and dimensions, and customize the docking box according to the characteristics of the target ligand. Finally, AutoDock Vina was utilized for molecular docking operations.

### 2.10 Quantitative real-time PCR analysis

RNA extraction from Caco-2 cells was performed using the TRIzol reagent (Catalog No. 15596026, Invitrogen, USA). The purity and concentration of the extracted RNA were precisely determined using a NanoDrop spectrophotometer (Agilent Technologies, USA). Subsequently, single-stranded complementary DNA (cDNA) was synthesized via reverse transcription. The HiScript II QRT SuperMix for qPCR (Product No. R223-01, Vazyme, China) was employed following the detailed instructions provided by the manufacturer. For gene expression relative quantification, qRT-PCR was performed using ChamQ SYBR Color qPCR Master Mix (Vazyme, China, No. Q431-02) with a Fast 7500 real-time PCR system (Applied Biosystems, USA). The 2^- ΔΔCT^ method calculated target gene fold-changes, with GAPDH as the endogenous reference for data normalization. The qRT-PCR primers, custom-synthesized by Ruibo Biotech (Guangzhou, China), for CaMK2D were:

Forward: 5’-CAACAGTCCCCATCAAGCCA-3’;

Reverse: 5’-GAAGACGTGGCACTGTTGAC-3’.

### 2.11 Plasmid construction and transient transfection

The CaMK2D expression plasmid was synthesized by Guangzhou Jintai Biotechnology Co., Ltd. Caco-2 cells were transfected with 2 μg of the CaMK2D expression plasmid or the control vector using Lipofectamine 3000 for 48 h. The transfection procedures were conducted according to the manufacturer’s recommended protocols.

### 2.12 WB

Immunoblotting was employed to measure the secretion levels of total CaMK2D (t-CaMK2D) and phosphorylated CaMK2D (p-CaMK2D). Samples were obtained from intestinal mucosa and Caco-2 cells. Intestinal tissues were collected and homogenized in a 4% paraformaldehyde solution. Caco-2 cells were seeded in 6-well plates at a density of 2×10^5^ cells per well. They were cultured in PBS under OGD conditions for 12 h, followed by incubation with or without RAPA (0.94 μg mL^-^¹) in complete medium for 2 h. The samples were treated with RIPA lysis buffer, and the BCA method was used to determine protein concentration. A total of 15 μg of the total protein sample was separated by electrophoresis on a 12.5% (w/v) SDS-PAGE gel and then transferred onto polyvinylidene fluoride membranes. The membranes were blocked with a 5% (w/v) fat-free dry milk solution. Subsequently, they were incubated with anti-β-actin (1:1000), t-CaMK2D antibody (1:1000), and p-CaMK2D antibody (1:1000). Afterward, the membranes were incubated with a peroxidase-conjugated secondary antibody (1:2000). Protein bands were detected using chemiluminescence reagents and analyzed with the Invitrogen iBright system (Thermo Fisher, USA).

### 2.13 Statistical analysis

**Figure 1:**
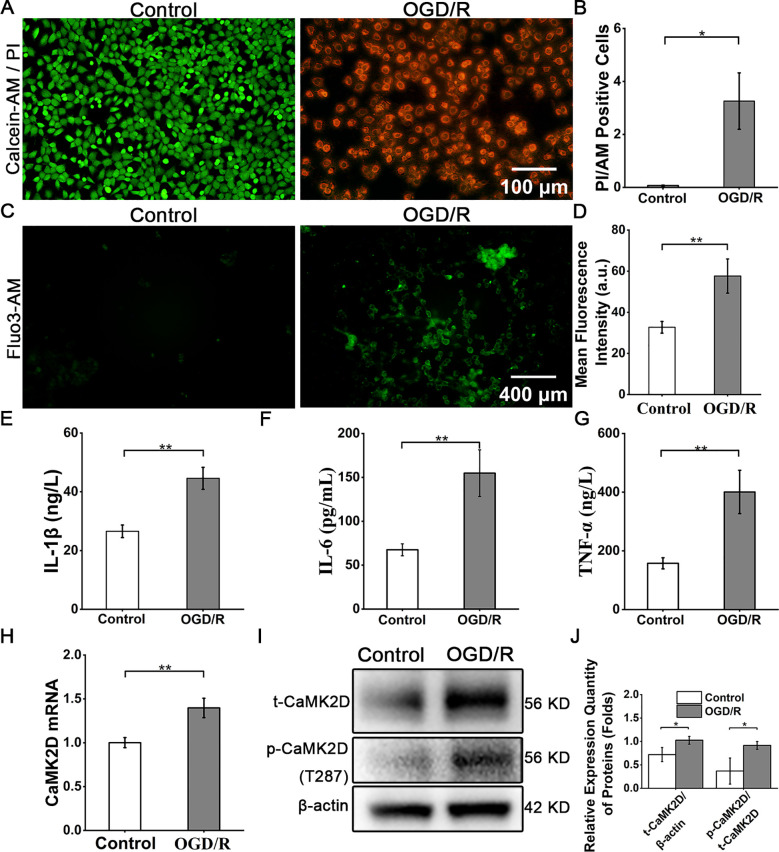
CaMK2D overexpression reversed protective effect of RAPA. **(A)** Representative images of cell live-dead staining (scale bar = 100 μm). **(B)** Quantification of cell viability. **(C)** Flow cytometry plots for apoptosis analysis. **(D)** Quantification of apoptotic cells. **(E)** Fluorescence intensity images of Fluo-3AM-loaded cell (scale bar = 400 μm). **(F)** Quantification of relative fluorescence intensity. ELISA results for the inflammatory cytokines **(G)** IL-1β, **(H)** IL-6, and **(I)** TNF-α in cell supernatants. **(J)** WB images for total CaMK2D (t-CaMK2D) and phosphorylated CaMK2D (p-CaMK2D). **(K)** Quantification of the relative expression of t-CaMK2D and **(L)** p-CaMK2D. Data are presented as means ± SD, n=3. Statistical tests: One-way ANOVA and non-parametric tests. *P < 0.05, **P < 0.01, ***P < 0.001.

The data were analyzed with IBM SPSS Statistics 26 software. Results were presented as mean ± standard deviation (SD). To compare the two groups, a two-tailed t-test was conducted. For multiple-group comparisons (e.g., **Figure 1**), one-way analysis of variance (one-way ANOVA) was used, followed by *post-hoc* tests as appropriate; non-parametric tests were applied when data did not meet the assumptions of parametric tests. Statistical significance was determined with p-values: *P* < 0.05 (*), *P* < 0.01 (**), *P* < 0.001 (***). Each experiment was replicated a minimum of three times. To ensure statistical power, three replicates were included in each experiment, and this setup was estimated to yield 80% power.

## 3 Results

### 3.1 I/R injury and CaMK2D upregulation in models

**Figure 2:**
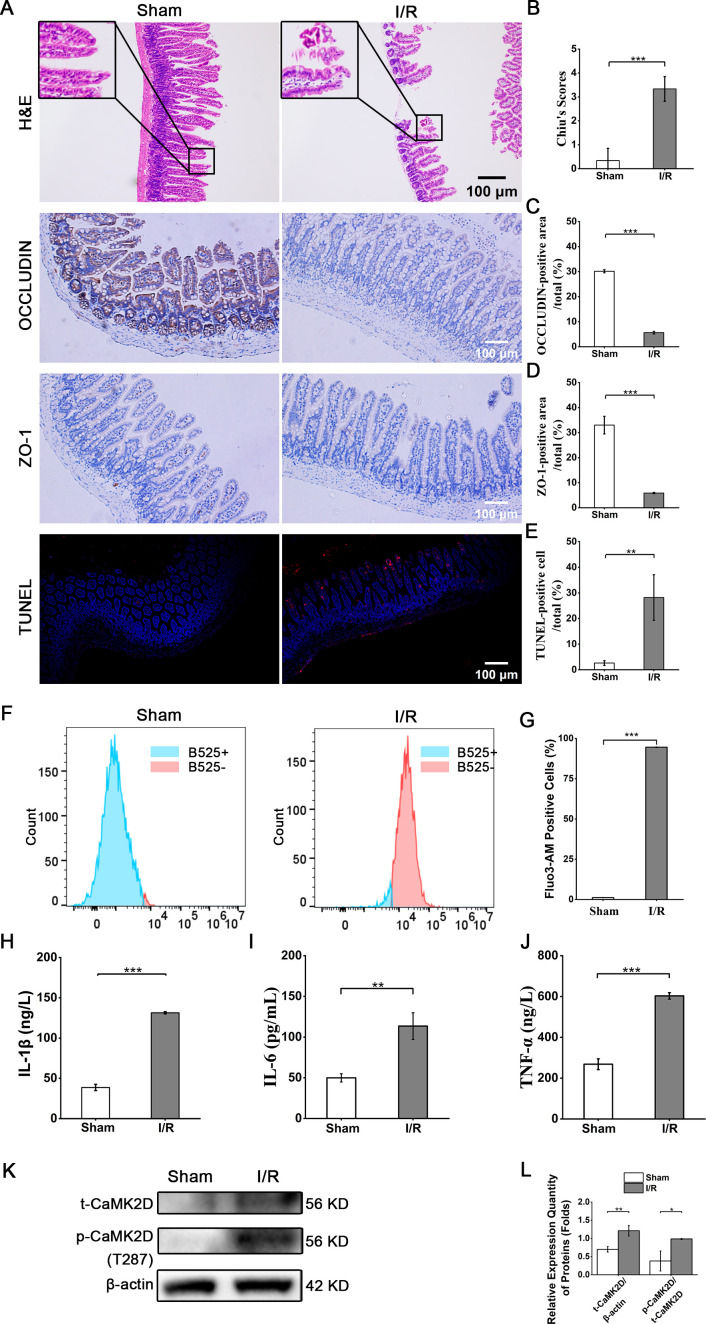
OGD/R-mediated cell death, inflammatory response, and CaMK2D upregulation and activation. **(A)** Representative images of cell live-dead staining (scale bar = 100 μm). **(B)** Quantification of cell viability. **(C)** Fluo3-AM staining for detecting intracellular calcium level (scale bar = 400 μm). **(D)** Quantification of intracellular calcium level. Representative images of ELISA results for the inflammatory cytokines **(E)** IL-1β, **(F)** IL-6, and **(G)** TNF-α in cell supernatants. **(H)** qRT-PCR results for CaMK2D mRNA expression. **(I)** WB images for total CaMK2D (t-CaMK2D) and phosphorylated CaMK2D (p-CaMK2D). **(J)** Quantification of the relative expression of t-CaMK2D and p-CaMK2D. Data are presented as means ± SD, n=3. Statistical test: Two-tailed t-test. *P < 0.05, **P < 0.01.

Systematic evaluation of intestinal mucosal injury and CaMK2D expression was performed in both *in vitro* (Caco-2 OGD/R) and *in vivo* (murine intestinal I/R) models. In Caco-2 OGD/R cultures, cell viability (Calcein-AM/Propidium Iodide (PI) live-dead staining assay, **Figures 2A, B**) decreased by 69.3 ± 7.2% compared to Control group (*P* < 0.05). Intracellular calcium overload confirmed by Fluo-3 AM imaging significantly rose in OGD/R group compared to the Control group (**Figures 2C, D**). Concomitantly, the release of inflammatory factors IL-1β, IL-6, and TNF-α was significantly augmented in the OGD/R-treated Caco-2 cells (**Figures 2E-G**). Regarding CaMK2D, qRT-PCR analysis confirmed the increase of CaMK2D at the mRNA level following OGD/R treatment (**Figure 2H**). WB analysis of the Caco-2 samples demonstrated a significant upregulation of both total CaMK2D (t-CaMK2D) and phosphorylated CaMK2D (p-CaMK2D, Thr287), indicating the detrimental role of p-CaMK2D (Thr287) in intestinal epithelial function (**Figures 2I, J**).

**Figure 3:**
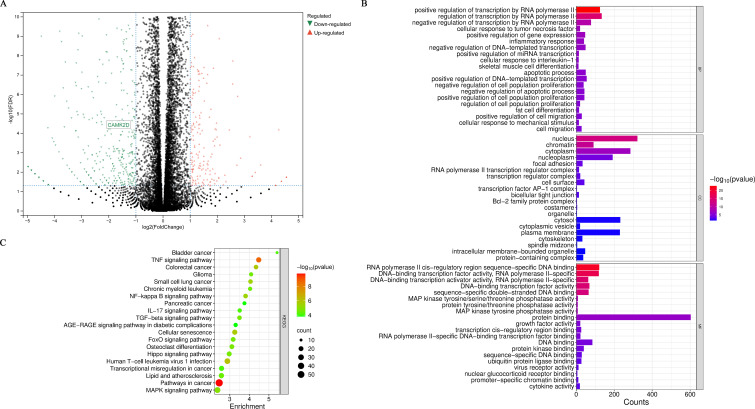
I/R-mediated intestinal mucosal injury and CaMK2D upregulation and activation. **(A)** Representative images of H&E, immunohistochemical and TUNEL staining (scale bar = 100 μm). Quantification of **(B)** Chiu’s scores, **(C)** OCCLUDIN, **(D)** ZO-1, and **(E)** TUNEL staining of treated mice. **(F)** Representative flow cytometry histograms of Fluo-3AM-loaded IECs for calcium detection. **(G)** Quantification of intracellular calcium concentration. ELISA results for the inflammatory cytokines **(H)** IL-1β, **(I)** IL-6, and **(J)** TNF-α in serum. **(K)** WB images for total CaMK2D (t-CaMK2D) and phosphorylated CaMK2D (p-CaMK2D). **(L)** Quantification of the relative expression of t-CaMK2D and p-CaMK2D. Data are presented as means ± SD, n=3. Statistical test: Two-tailed t-test. *P < 0.05, **P < 0.01, ***P < 0.001.

In the animal intestinal I/R model, histological analysis of the terminal ileal tissue using H&E staining revealed extensive mucosal injury. The intestinal villi in the I/R group showed severe damage, characterized by vacuolar degeneration and disorganization of the mucosal architecture. Employing the Chiu’s scoring system for quantification, the I/R group exhibited a significantly higher score, indicating more severe mucosal injury compared to the sham group (**Figures 3A, B**). In addition, the immunohistochemical staining of OCCLUDIN and ZO-1 proteins revealed the integrity of the intestinal mucosal barrier was impaired in the I/R group compared to the Sham group (**Figures 3A-D**). Moreover, TUNEL staining indicated that the proportion of apoptotic cells in the I/R group was significantly higher than that in the Sham group (**Figures 3A-E**). Intracellular calcium concentration increased significantly following intestinal I/R treatment, with the level much higher than the normal state, indicating calcium overload (**Figures 3F, G**). Parallelly, the levels of cytokines, specifically IL-1β, IL-6, and TNF-α were significantly elevated after intestinal I/R treatment (**Figures 3H-J**). With respect to CaMK2D, WB analysis of the intestinal mucosal tissue samples revealed that both total CaMK2D (t-CaMK2D) and phosphorylated CaMK2D (p-CaMK2D) were significantly upregulated (**Figures 3K, L**).

### 3.2 RNA-seq unveils OGD/R-induced CaMK2D upregulation and MCODE analysis of DEGs

**Figure 4:**
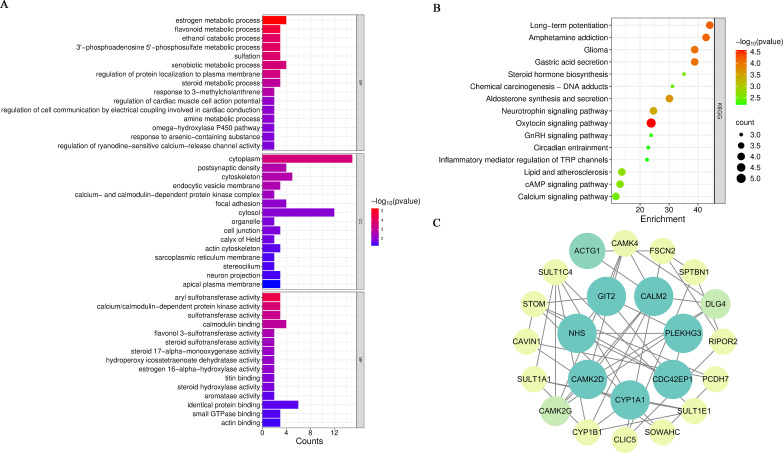
Global analysis of differentially expressed genes (DEGs) in Caco-2 cells subjected to OGD/R with and without RAPA treatment. **(A)** Volcano plot visualizing the DEGs. Genes with log FC ≥ 1 and FDR < 0.01 are shown in red (upregulated), and those with log FC ≤ -1 and FDR < 0.01 are in green (downregulated). This plot provides an overview of the magnitude and significance of gene expression changes. A total of 816 DEGs were identified, with 215 upregulated and 601 downregulated genes. **(B)** Gene Ontology (GO) functional enrichment analysis results for the 816 DEGs. **(C)** Kyoto Encyclopedia of Genes and Genomes **(KEGG)** pathway enrichment analysis of the 816 DEGs.

To investigate the molecular mechanisms underlying RAPA’s protective effects against intestinal I/R injury (supported by our previous study and relevant literature (10, 11, 18)),we performed RNA-Seq on Caco-2 cells exposed to OGD/R injury with and without RAPA treatment to identify differentially expressed genes (DEGs) and investigate potential molecular pathways underlying RAPA’s protective effects. EdgeR was applied for differential expression analysis of the RNA-Seq date. The screening criteria were set as follows: genes with log FC ≥ 1 and FDR < 0.01 were considered upregulated, while those with log FC ≤ - 1 and FDR < 0.01 were regarded as downregulated. This identified 816 DEGs, comprising 215 upregulated and 601 downregulated genes. The DEGs were then visualized in a volcano plot, providing an intuitive overview of the gene expression changes (**Figure 4A**). Gene Ontology (GO) functional enrichment analysis (**Figure 4B**) revealed predominant biological processes (BP) enrichments in transcriptional regulation, inflammatory response, cell proliferation, and apoptosis (e.g., “positive regulation of transcription by RNA polymerase II,” “inflammatory response,” and “regulation of cell population proliferation”). Cellular components (CC) analysis highlighted significant enrichment in nucleus, cytoplasmic, and chromatin-associated structures, suggesting these compartments as key regulatory sites during I/R injury. Molecular function (MF) enrichment identified DNA binding, transcription factor activity, and protein structure (**Figure 4B**). This association underscored the crucial roles of these genes in transcription and protein-protein interactions (PPI), which are integral to the complex molecular mechanisms underlying I/R injury. Kyoto Encyclopedia of Genes and Genomes (KEGG) pathway analysis demonstrated enrichment of pathways closely related to cancer, immunity, and cell signal transduction. Notable pathways included “Pathways in cancer,” “TNF signaling pathway,” and “NF-kappa B signaling pathway” (**Figure 4C**). These pathways may represent critical therapeutic targets in the pathogenesis of intestinal I/R injury.

Systemic clustering of DEGs was performed using the MCODE algorithm to explore functional modules. Top five clusters (Cluster 1-5) by MCODE scores were selected for in-depth investigation based on their distinct GO enrichments, KEGG pathways associations, and PPI network characteristics, providing insights to molecular mechanisms during I/R injury.

#### 3.2.1 Cluster 1

Cluster 1 consists of 56 genes. GO functional enrichment (**Supplementary Figure 1A**) revealed Cluster 1 was predominantly associated with BP linked to transcriptional regulation and cell-cycle control, critical for cellular homeostasis but frequently disrupted in I/R injury. CC primarily included the nucleus and chromatin, key sites for DNA storage and gene expression regulation. MF highlighted DNA binding and transcription factor activity, underscoring their regulatory roles in the regulation of gene expression. KEGG pathway analysis identified enrichment in cancer-related pathways (“Pathways in cancer”), infection pathways (“Human T-cell leukemia virus 1 infection”), and inflammatory pathways, suggesting links between cellular stress responses, immune activation, and oncogenic transformation in I/R injury (**Supplementary Figure 1B**). PPI network analysis identified FOS and WNT1 as core nodes, potentially serving as central regulators of these processes associated with this cluster (**Supplementary Figure 1C**).

#### 3.2.2 Cluster 2

Cluster 2 consists of 28 genes. Cluster 2 exhibited BP enrichment in transcriptional regulation, cell proliferation, migration, and inflammation processes central to tissue repair but potentially pathogenic when dysregulated. CC analysis predominantly identified the extracellular region, crucial for intercellular communication. MF revealed enrichment in growth factor activity, chemokine signaling, and protein binding, indicating involvement in cell signaling regulation (**Supplementary Figure 2A**). KEGG pathways showed significant enrichment in TNF and NF-κB signaling alongside cancer-related pathways, implying dual rols in inflammation and oncogenesis during I/R injury (**Supplementary Figure 2B**). IRF1 emerged as a pivotal PPI hub, suggesting its critical role in coordinating cluster-specific biological functions (**Supplementary Figure 2C**).

#### 3.2.3 Cluster 3

Cluster 3 consists of 10 genes. Cluster 3 demonstrated BP enrichment in cell division and DNA metabolism processes, including replication and repair, essential for genomic integrity. CC analysis focused on mitotic structures such as the spindle apparatus and the centrosome, responsible for chromosomal segregation. MF analysis highlighted ATP hydrolysis, microtubule dynamics, and DNA unwinding, integral to mitotic machinery function (**Supplementary Figure 3A**). KEGG pathway analysis revealed exclusive enrichment in the “Motor proteins” pathway, suggesting specialized roles in intracellular transport and motility. PPI network analysis identified DEPDC1 and RTEL1 as key regulators, potentially coordinating cell division and DNA metabolic processes (**Supplementary Figure 3B**).

#### 3.2.4 Cluster 4

Cluster 4 consists of 21 genes. Cluster 4’BP enrichment centered on transcriptional regulation, inflammatory response, and angiogenesis processes vital for post-ischemic tissue repair. CC analysis predominantly identified nuclear and chromatin-associated structures, central to stress-induced gene expression. MF analysis emphasized DNA binding and transcription factor activity, linked to cellular stress responses (**Supplementary Figure 4A**). KEGG pathways showed enrichment in inflammation, viral infection, and tumorigenesis pathways, suggesting roles in immune activation response, antiviral defenses, and oncogenic potential during I/R injury (**Supplementary Figure 4B**). GATA6 and ZC3H12A emerged as PPI hubs, likely orchestrating cluster-specific biological activities (**Supplementary Figure S4C**).

#### 3.2.5 Cluster 5

**Figure 5:**
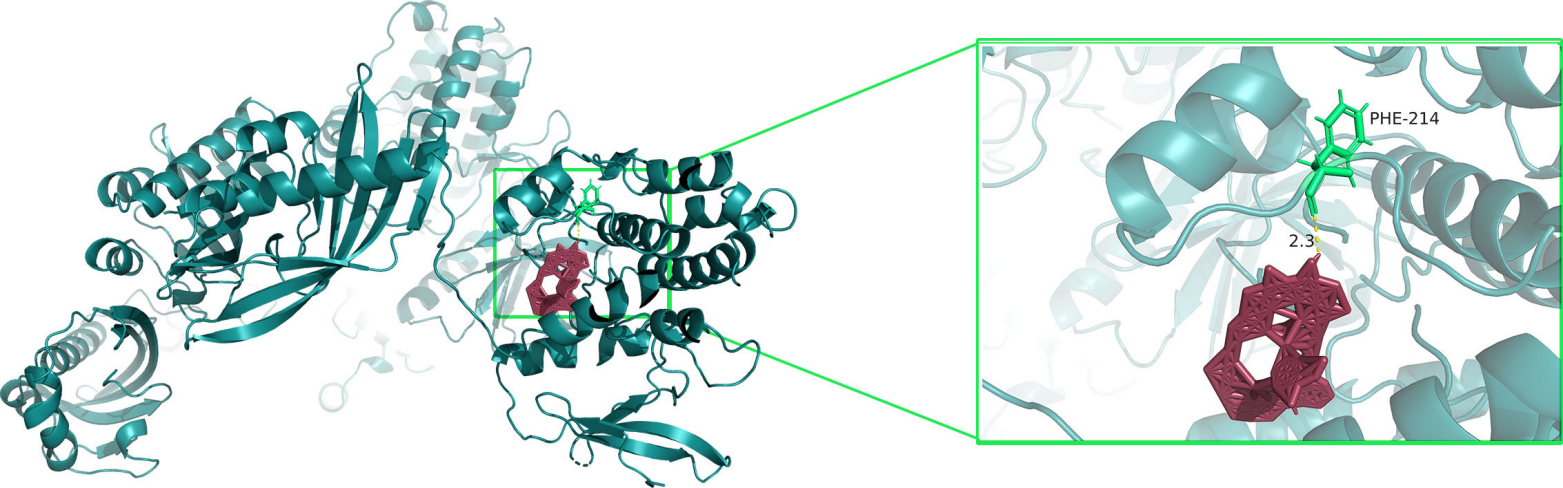
GO functional enrichment, KEGG pathway, and PPI network analysis of Cluster 5. **(A)** GO functional enrichment analysis of Cluster 5. **(B)** KEGG pathway enrichment analysis of Cluster 5. **(C)** Protein-protein interaction network of Cluster 5.

Cluster 5 consists of 23 genes. Cluster 5 exhibited BP enrichment in metabolic processes (e.g., “estrogen metabolic process”) and calcium signaling regulation (e.g., “regulation of ryanodine-sensitive calcium-release channel activity”, “calcium- and calmodulin-dependent protein kinase complex”, “calcium/calmodulin-dependent protein kinase activity”), with aberrant calcium handing directly linked to intestinal I/R injury pathophysiology. CC analysis highlighted calcium-regulatory structures and cytoskeletal components, critical for maintaining cellular integrity during stress. MF analysis focused on calcium-dependent enzyme activities and metabolic regulation, directly influencing calcium signaling and metabolic homeostasis (**Figure 5A**). KEGG pathway analysis identified the calcium signaling pathway as primary enrichment, alongside less prominent pathways (e.g., long-term potentiation), potentially reflecting indirect nervous system modulation during injury (**Figure 5B**). PPI network analysis positioned CaMK2D as a central regulator (**Figure 5C**). This calcium/calmodulin-dependent kinase has been implicated in myocardial I/R injury prognosis and may drive inflammatory responses via calcium signaling dysregulation in intestinal I/R injury. Given Cluster 5’s alignment with key I/R injury mechanisms (calcium overload and inflammatory response), as well as the potential core regulatory role of CaMK2D, this cluster warrants prioritized investigation in elucidate RAPA’s therapeutic mechanisms in intestinal I/R injury.

### 3.3 Molecular docking analysis of RAPA-CaMK2D interaction

**Figure 6:**
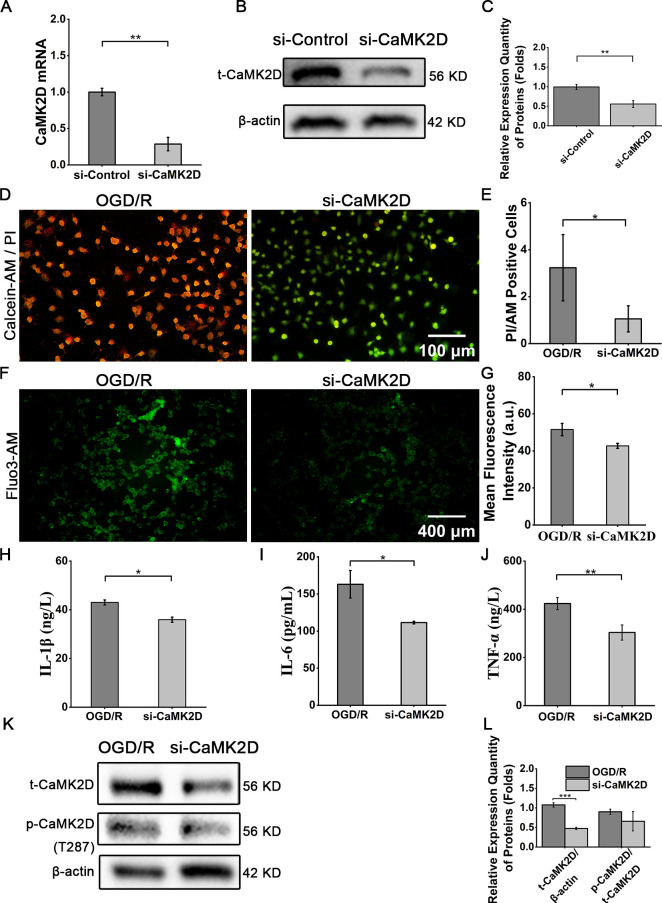
Molecular docking of RAPA and CaMK2D.

Molecular docking simulations were performed using AutoDock Vina to evaluate the binding interaction between RAPA and CaMK2D. The three-dimensional (3D) structure of CaMK2D (PDB ID: D6R938) was retrieved from the Protein Data Bank. Prior to docking, RAPA underwent energy minimization and Gasteiger charge assignment to optimize its electrostatic properties. The binding analysis revealed a binding energy of -12.1 kcal/mol (**Table 1**), indicating strong binding affinity between RAPA and CaMK2D. This value aligns with established thresholds for significant molecular interactions in protein-ligand studies. Visual inspection of the docking conformation (**Figure 6**) identified phenylalanine 214 (PHE-214) as the critical binding residue, with a hydrogen bond (H-bond) distance of 2.3 Å between RAPA and the aromatic ring of PHE-214. The H-bond network, represented by dotted lines in the interaction diagram, suggests stabilizing interactions critical for complex formation.

**Table 1:** Molecular docking results of the rapamycin and CaMK2D.

Molecular name	Target name	PDB/AlphaFold ID	Score (kcal/mol)	Center x	Center y	Center z	Size x	Size y	Size z
RAPA	CaMK2D	8uso	-12.1	55.0	162.4	0.5	98.4	90.5	145.2

### 3.4 Inhibition of CaMK2D alleviated intestinal I/R injury

**Figure 7:**
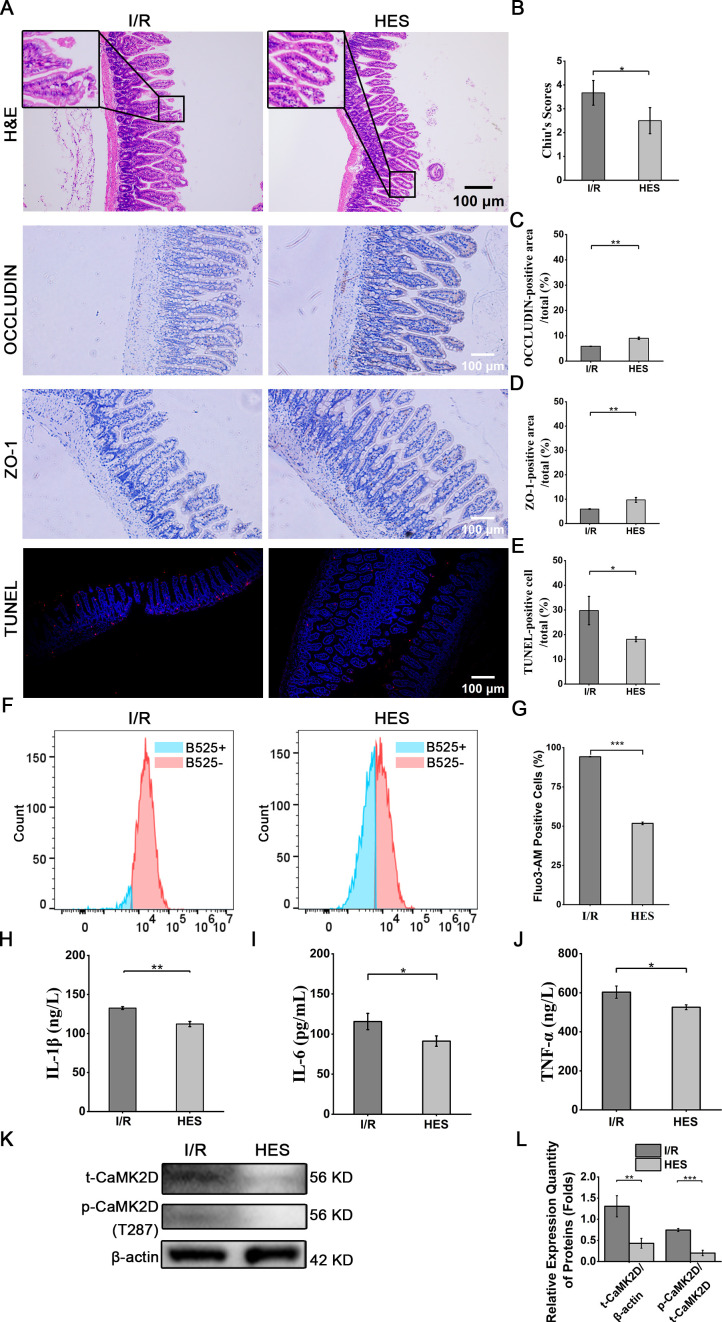
Knockdown of CaMK2D alleviated OGD/R injury. **(A)** qRT-PCR results for CaMK2D mRNA expression. **(B)** WB images for total CaMK2D (t-CaMK2D). **(C)** Quantification of the relative expression of t-CaMK2D. **(D)** Representative images of cell live-dead staining (scale bar = 100 μm). **(E)** Quantification of cell viability. **(F)** Fluo3-AM staining for detecting intracellular calcium level (scale bar = 400 μm). **(G)** Quantification of intracellular calcium level. ELISA results for the inflammatory cytokines **(H)** IL-1β, **(I)** IL-6, and **(J)** TNF-α in cell supernatants. **(K)** WB images for total CaMK2D (t-CaMK2D) and phosphorylated CaMK2D (p-CaMK2D). **(L)** Quantification of the relative expression of t-CaMK2D and p-CaMK2D. Data are presented as means ± SD, n=3. Statistical test: Two-tailed t-test. *P < 0.05, **P < 0.01.

To validate the functional role of CaMK2D in OGD/R injury, Caco-2 cells were transfected with siRNA targeting CaMK2D. Transfection efficiency was confirmed via qRT-PCR and WB, demonstrating a remarkable reduction in CaMK2D mRNA (71.4 ± 9.3% decrease) and protein levels (44.0 ± 8.9% decrease) compared to OGD/R groups (**Figures 7A-C**). Following OGD/R treatment, si-CaMK2D-transfected Caco-2 cells exhibited lower cell death (67.4 ± 17.3% decrease, *P* < 0.05) versus OGD/R groups, accompanied by 17.2% reduction in intracellular calcium level (Fluo 3-AM assay) and 16.5%/31.7%/28.4% decrease in IL-1β/IL-6/TNF-α secretion (ELISA) (**Figures 7D-J**). WB further confirmed 50.3% suppression of total CaMK2D (t-CaMK2D) expression in the si-CaMK2D cells (**Figures 7K, L**).

**Figure 8:**
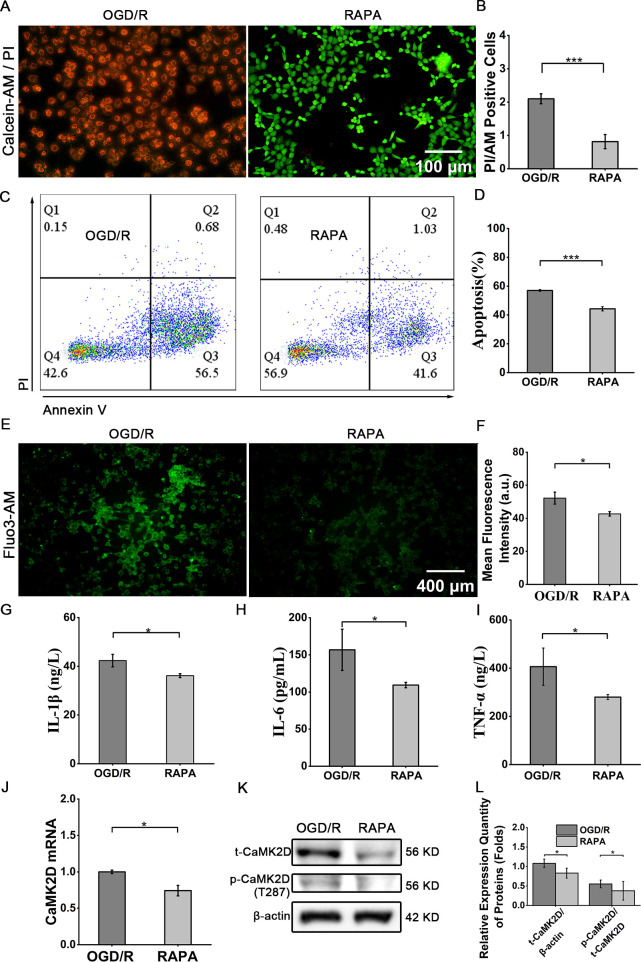
Inhibition of CaMK2D alleviated intestinal I/R injury. **(A)** Representative images of H&E, immunohistochemical and TUNEL staining (scale bar = 100 μm). Quantification of **(B)** Chiu’s scores, **(C)** OCCLUDIN, **(D)** ZO-1, and **(E)** TUNEL staining of treated mice. **(F)** Representative flow cytometry histograms of Fluo-3AM-loaded intestinal cells for calcium detection. **(G)** Quantification of intracellular calcium concentration. ELISA results for the inflammatory cytokines **(H)** IL-1β, **(I)** IL-6, and **(J)** TNF-α in serum. **(K)** WB images for total CaMK2D (t-CaMK2D) and phosphorylated CaMK2D (p-CaMK2D). **(L)** Quantification of the relative expression of t-CaMK2D and p-CaMK2D. Data are presented as means ± SD, n=3. Statistical test: Two-tailed t-test. *P < 0.05, **P < 0.01, ***P < 0.001.

HES (5 mg/kg) a specific CaMK2D inhibitor (5 μg/kg, i.p.), was administered intraperitoneally daily for 4 days prior to intestinal I/R injury (15). Histological analysis revealed 31.8% lower Chiu’s scores (*P* < 0.05) and 1.5-fold/1.6-fold higher OCCLUDIN/Zo-1 expression (IHC) in HES-treated mice versus I/R controls (**Figures 8A, E-D**). TUNEL staining demonstrated 39.2% reduction in apoptotic cells (*P* < 0/05), while Fluo-3 fluorescence intensity (indicating intracellular calcium ion concentration) decreased by 45.1% in IECs (flow cytometry) (**Figures 8A-G**). Serum inflammatory cytokines (IL-1β/IL-6/TNF-α) showed 12.8-21.1% decreases in HES-treated mice (**Figures 8H-J**). WB confirmed parallel reductions in t-CaMK2D (67.0%) and p-CaMK2D (73%) levels in HES-treated animals (**Figures 8K, L**).

### 3.5 RAPA reversed intestinal I/R injury by down-regulating CaMK2D expression and phosphorylation

**Figure 9:**
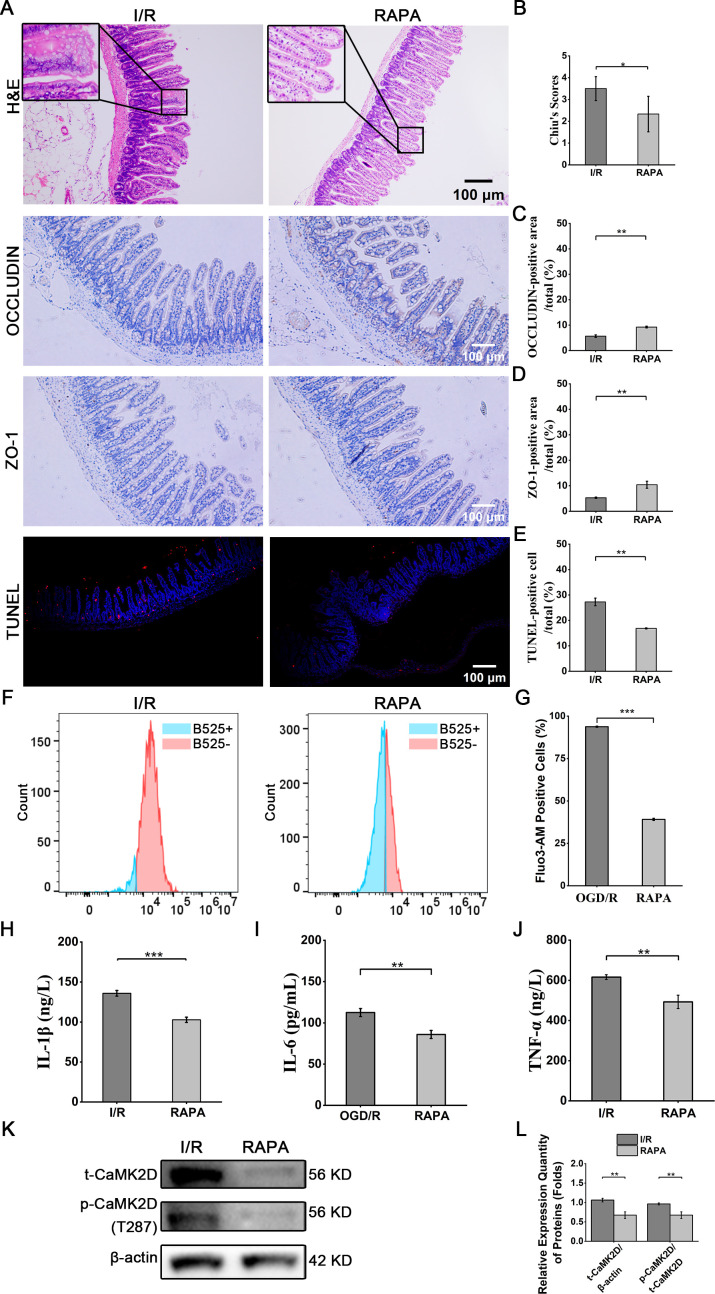
RAPA reversed OGD/R injury by inhibiting CaMK2D expression and phosphorylation. **(A)** Representative images of cell live-dead staining (scale bar = 100 μm). **(B)** Quantification of cell viability. **(C)** Flow cytometry plots for apoptosis analysis. **(D)** Quantification of apoptotic cells. **(E)** Fluorescence intensity images of Fluo-3AM-loaded cell (scale bar = 400 μm). **(F)** Quantification of relative fluorescence intensity. ELISA results for the inflammatory cytokines **(G)** IL-1β, **(H)** IL-6, and **(I)** TNF-α in cell supernatants. **(J)** qRT-PCR results for CaMK2D mRNA expression. **(K)** WB images for total CaMK2D (t-CaMK2D) and phosphorylated CaMK2D (p-CaMK2D). **(L)** Quantification of the relative expression of t-CaMK2D and p-CaMK2D. Data are presented as means ± SD, n=3. Statistical test: Two-tailed t-test. *P < 0.05, ***P < 0.001.

To investigate the therapeutic mechanism of RAPA in OGD/R injury, Caco-2 cells pre-treated with OGD were co-incubated with RAPA for 2 hours. Cell viability (live-dead staining, **Figure 9B**) and apoptosis (flow cytometry, **Figure 9D**) were significantly improved in RAPA-treated groups compared to OGD/R controls (viability: 32.3 ± 1.6% vs 55.7 ± 6.8%, *P* < 0.001; apoptosis: 57.0 ± 0.19% vs 44.3 ± 1.5%, *P* < 0.001) (**Figures 9A-D**). RAPA treatment reduced intracellular calcium overload (Fluo-3 AM assay: 52.2 ± 3.6 vs 42.7 ± 1.3) and suppressed inflammatory cytokine secretion (IL-1β: 42.3 ± 2.6 ng/L vs 36.2 ± 0.8 ng/L; IL-6: 156.8 ± 27.8 pg/mL vs 109.4 ± 3.7 pg/mL; TNF-α: 406.3 ± 77.3 ng/L vs 280.2 ± 10.2 ng/L; *P* < 0.05) (**Figures 9E-I**). qRT-PCR and WB demonstrated 25.8% reduction in CaMK2D mRNA (*P* < 0.05) and 49.0% decrease in t-CaMK2D protein levels (*P* < 0.05). p-CaMK2D levels showed 54.5% inhibition (**Figures 9J-L**).

**Figure 10:**
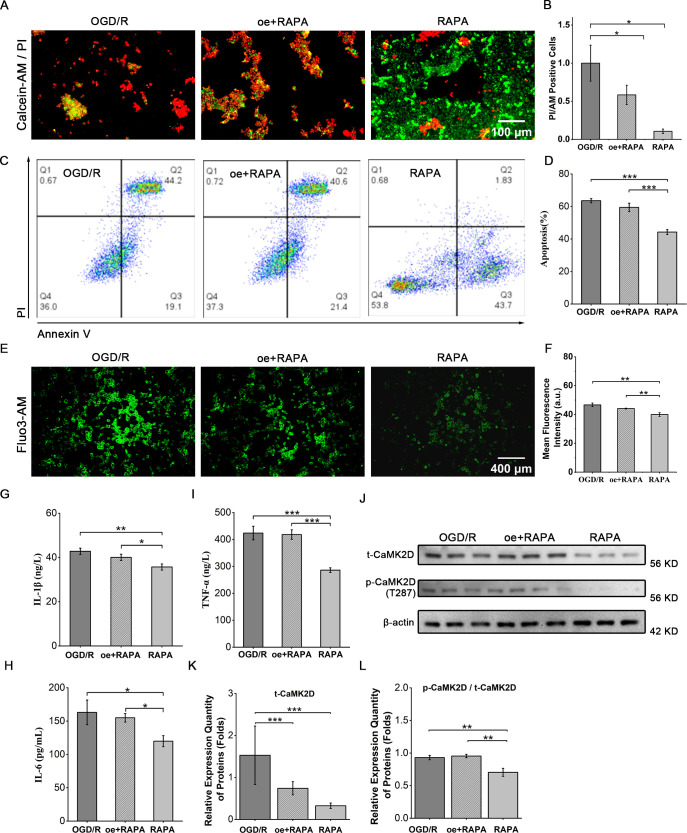
RAPA reversed I/R injury by inhibiting CaMK2D expression and phosphorylation. **(A)** Representative images of H&E, immunohistochemical and TUNEL staining (scale bar = 100 μm). Quantification of **(B)** Chiu’s scores, **(C)** OCCLUDIN, **(D)** ZO-1, and **(E)** TUNEL staining of treated mice. **(F)** Representative flow cytometry histograms of Fluo-3AM-loaded intestinal cells for calcium detection. **(G)** Quantification of intracellular calcium concentration. ELISA results for the inflammatory cytokines **(H)** IL-1β, **(I)** IL-6, and **(J)** TNF-α in serum. **(K)** WB images for total CaMK2D (t-CaMK2D) and phosphorylated CaMK2D (p-CaMK2D). **(L)** Quantification of the relative expression of t-CaMK2D and p-CaMK2D. Data are presented as means ± SD, n=3. Statistical test: Two-tailed t-test. *P < 0.05, **P < 0.01, ***P < 0.001.

RAPA administration (1.5 mg kg^-^¹) at 2 hours post-reperfusion significantly improved intestinal histology (Chiu’s score: 2.3 ± 0.8 vs 3.5 ± 0.5 in I/R controls, *P* < 0.05) (**Figure 10A** and 9B). OCCLUDIN and ZO-1 expression increased 1.6-fold and 1.9-fold (IHC) in RAPA-treated mice (*P* < 0.01) (**Figures 10A-D**). TUNEL staining revealed 38.2% ± 0.7% reduction in apoptotic cells (P < 0.01) (**Figures 10A, E**). Flow cytometry showed 58.3 ± 0.7% lower intracellular calcium levels (Fluo-3 AM: 93.8 ± 0.36% vs 39.1 ± 0.6%; *P* < 0.001) (**Figures 10F, G**). Serum inflammatory cytokines (IL-1β/IL-6/TNF-α) in RAPA-treated mice decreased by 20.0-24.4% (*P* < 0.01) (**Figures 10H-I**). WB confirmed 36.5 ± 8.0% and 30.0± 8.9% reductions in t-CaMK2D and p-CaMK2D levels in intestinal tissues (*P* < 0.01) (**Figures 10K, L**).

### 3.6 Overexpression of CaMK2D antagonized RAPA-mediated protective effects

To investigate whether overexpression of CaMK2D can reserve the therapeutic effect of RAPA, we transfected Caco-2 cells with a CaMK2D overexpression plasmid. After screening different plasmid concentrations, a concentration of 2 μg per well in 6-well plates was selected for subsequent experiments (**Supplementary Figure 5**). Overexpression of CaMK2D significantly reversed the rescuing effects of RAPA on cell viability (live-dead staining) and apoptosis (flow cytometry) (**Figures 1A-D**). Additionally, CaMK2D overexpression exacerbated intracellular calcium overload and the level of inflammation in RAPA-treated group (**Figures 1E-I**). WB analysis further confirmed that overexpression of CaMK2D effectively abrogated the therapeutic effect of RAPA (**Figures 1J-L**).

## 4 Discussion

In this study, we initially demonstrated that both OGD/R and intestinal I/R could induce severe damage, including cell deaths, apoptosis, disruption of tight junctions, calcium overload, and inflammatory response. Notably, while prior studies reported CaMK2D activation in non-intestinal I/R models (e.g., myocardial, neuronal I/R) (19, 20), this study is the first to demonstrate that CaMK2D hyperactivation serves as a central pathological node mediating intestinal I/R injury, distinct from its role in other organs. Core evidence confirming this includes: RNA-Seq and MCODE clustering identified CaMK2D as the core of a calcium signaling module (Cluster 5) linked to I/R pathophysiology; molecular docking verified direct binding between RAPA and CaMK2D (binding energy: -12.1 kcal/mol, critical residue: PHE-214); genetic (siRNA) and pharmacological (HES) inhibition of CaMK2D alleviated injury; and CaMK2D overexpression abrogated RAPA’s protective effects. Collectively, these findings confirm that RAPA alleviates intestinal I/R injury by suppressing CaMK2D expression and phosphorylation (**Scheme 1**).

Intestinal I/R injury involves interconnected pathological pathways, including energy metabolism disorders, mitochondrial damage (21–24),oxidative stress (25, 26), cytosolic calcium overload (27, 28), and neutrophil extracellular traps (NETs) mediated injury (29), all of which contribute to intestinal epithelial damage and barrier dysfunction. Notably, calcium overload is a central upstream trigger of these pathological cascades, yet the key regulatory kinases linking calcium dyshomeostasis to intestinal I/R injury remain incompletely defined.

The intracellular calcium ion signaling pathway serves as a master regulator of cellular hoeostasis (30), with diverse biological functions including synaptic transmission (31, 32) and cytoskeletal remodeling (33). However, in the context of intestinal I/R injury, its core role lies in maintaining epithelial integrity via tight junction regulation and mediating inflammatory responses by promoting inflammatory cell activation and cytokine release (34, 35)—two processes central to intestinal epithelial damage. As a calcium/calmodulin-dependent kinase, CaMK2D acts as a critical mediator of this pathological cascade. Clarifying how CaMK2D modulates calcium signaling and interacts with RAPA is therefore essential to unraveling the mechanism of intestinal I/R injury and developing targeted therapies.

As a key calcium/calmodulin-dependent kinase, CaMK2D participates in normal intracellular signal transduction, but its abnormal activation during intestinal I/R injury triggers a pathological cascade (19, 20, 36, 37). At the cellular level, excessive CaMK2D activation disrupts intestinal epithelial tight junction structure (38, 39), inducing abnormal phosphorylation and reduced membrane localization of OCCLUDIN and ZO-1 to impair barrier integrity and increase intestinal permeability (40, 41)—a process that facilitates harmful substance translocation and further tissue damage (42, 43). The activation of CaMK2D is also closely related to the increase in apoptosis (44). It may induce the death of more intestinal cells by regulating the expression or activity of apoptosis-related proteins, thereby affecting the normal structure and function of intestinal tissues (45). On one hand, after CaMK2D is activated, it may activate pro-apoptotic proteins, inhibit the function of anti-apoptotic proteins, activate the intracellular apoptotic signaling pathway, and initiate the apoptosis program (46). Concurrently, CaMK2D mediates inflammatory signaling pathway conduction, driving massive release of pro-inflammatory cytokines and cascade amplification of local inflammation (47–49). In terms of the inflammatory response, the activation of CaMK2D may be involved in the conduction of inflammatory signaling pathways, leading to the massive release of inflammatory factors, exacerbating the local inflammatory response, forming an inflammatory cascade amplification effect, disrupting the microenvironment of the intestinal tissues, and interfering with the normal physiological functions of the intestine (50). Collectively, CaMK2D hyperactivation integrates barrier dysfunction, apoptosis, and inflammation to promote intestinal I/R injury pathogenesis, highlighting its potential as a targeted therapeutic node.

RAPA’s specific inhibition of CaMK2D carries notable clinical relevance for intestinal I/R injury management—by suppressing CaMK2D, RAPA preserves intestinal barrier integrity and reduces inflammatory cytokine release, directly addressing the key pathological drivers of complications like MODS (3, 5). This extends beyond RAPA’s known roles in autophagy or mTOR signaling (10, 11), highlighting a novel therapeutic mechanism centered on CaMK2D targeting. Nevertheless, clinical translation of RAPA for intestinal I/R injury faces practical challenges, including its immunosuppressive side effects, optimal dosage determination, and definition of the therapeutic time window (18, 51, 52), which require further validation in large-scale preclinical and clinical studies to optimize application protocols.

In summary, CaMK2D plays a pivotal role in intestinal I/R injury. The activation of CaMK2D triggers pathological changes such as impairment of intestinal barrier integrity, increased apoptosis, and exacerbation of the inflammatory response. By inhibiting the activity of CaMK2D, RAPA has emerged as a potentially effective drug for the treatment of intestinal I/R injury. Nevertheless, in order to achieve better therapeutic outcomes, it is of great importance to conduct in-depth investigations into its mechanism of action and evaluate the feasibility of its clinical application.

## 5 Conclusion

This research investigated the role of CaMK2D in intestinal I/R injury and the therapeutic efficacy of RAPA. Both *in vitro* and *in vivo* models indicated that elevated CaMK2D expression was positively linked to injury severity. Inhibition of CaMK2D significantly mitigated the injury, and vice versa. RAPA, by downregulating CaMK2D, effectively exerted a protective effect. Evidently, CaMK2D is a critical determinant in intestinal I/R injury, and RAPA emerges as a promising therapeutic option for managing this condition.

## Data Availability

The data presented in the study are deposited in the NCBI repository, accession number PRJNA1456886.

## References

[1] Ikeda H Suzuki Y Suzuki M Koike M Tamura J Tong J . Apoptosis is a major mode of cell death caused by ischemia and ischemia/reperfusion injury to the rat intestinal epithelium. Gut. (1998) 42:530–7. doi: 10.1136/gut.42.4.530, PMID: 9616316 PMC1727054

[2] Lin D Zhang Y Wang S Zhang H Gao C Lu F . Ganoderma lucidum polysaccharide peptides GL-PPSQ2 alleviate intestinal ischemia-reperfusion injury via inhibiting cytotoxic neutrophil extracellular traps. Int J Biol Macromolecules. (2023) 244:125370. doi: 10.1016/j.ijbiomac.2023.125370, PMID: 37330081

[3] Li Y Cao Y Xiao J Shang J Tan Q Ping F . Inhibitor of apoptosis-stimulating protein of p53 inhibits ferroptosis and alleviates intestinal ischemia/reperfusion-induced acute lung injury. Cell Death Differ. (2020) 27:2635–50. doi: 10.1038/s41418-020-0528-x, PMID: 32203170 PMC7429834

[4] Huang Y . E3 ligase TRIM65 alleviates intestinal ischemia/reperfusion injury through inhibition of TOX4-mediated apoptosis. Cell Death Dis. (2024) 15:29. doi: 10.1038/s41419-023-06410-x, PMID: 38212319 PMC10784301

[5] Bala M Catena F Kashuk J De Simone B Gomes CA Weber D . Acute mesenteric ischemia: updated guidelines of the World Society of Emergency Surgery. World J Emerg Surg. (2022) 17:54. doi: 10.1186/s13017-022-00443-x, PMID: 36261857 PMC9580452

[6] Tan Y-Q Zhang W Xie Z-C Li J Chen H-W . CaMK II in cardiovascular diseases, especially caMK II-δ: friends or enemies. DDDT. (2024) 18:3461–76. doi: 10.2147/DDDT.S473251, PMID: 39132626 PMC11314529

[7] Hua Y Qian J Cao J Wang X Zhang W Zhang J . Ca2+/calmodulin-dependent protein kinase II regulation by inhibitor of receptor interacting protein kinase 3 alleviates necroptosis in glycation end products-induced cardiomyocytes injury. IJMS. (2022) 23:6988. doi: 10.3390/ijms23136988, PMID: 35805993 PMC9266390

[8] Ling H Gray CBB Zambon AC Grimm M Gu Y Dalton N . Ca^2+^ /calmodulin-dependent protein kinase II δ Mediates myocardial ischemia/reperfusion injury through nuclear factor-κB. Circ Res. (2013) 112:935–44. doi: 10.1161/CIRCRESAHA.112.276915, PMID: 23388157 PMC3673710

[9] Kong L Xiong F Sun N Xu C Chen Y Yang J . CaMKIIδ inhibition protects against myocardial ischemia/reperfusion injury: Role of Beclin-1-dependent autophagy. Eur J Pharmacol. (2020) 886:173539. doi: 10.1016/j.ejphar.2020.173539, PMID: 32918874

[10] Sheng R Wang W Zeng W Li B Yu H Li X . Macrophage membrane coated manganese dioxide nanoparticles loaded with rapamycin alleviate intestinal ischemia-reperfusion injury by reducing oxidative stress and enhancing autophagy. IJN. (2025) 20:3541–57. doi: 10.2147/IJN.S507546, PMID: 40125428 PMC11929519

[11] Xu Y Ou J Zhang C Chen J Chen J Li A . Rapamycin promotes the intestinal barrier repair in ulcerative colitis via the mTOR/PBLD/AMOT signaling pathway. Biochim Biophys Acta (BBA) - Mol Basis Dis. (2024) 1870:167287. doi: 10.1016/j.bbadis.2024.167287, PMID: 38862095

[12] Karakus IS Catak MC Frohne A Bayram Catak F Yorgun Altunbas M Babayeva R . Rapamycin controls lymphoproliferation and reverses T-cell responses in a patient with a novel STIM1 loss-of-function deletion. J Clin Immunol. (2024) 44:94. doi: 10.1007/s10875-024-01682-0, PMID: 38578569 PMC10997552

[13] Li Y Wen S Yao X Liu W Shen J Deng W . MicroRNA-378 protects against intestinal ischemia/reperfusion injury via a mechanism involving the inhibition of intestinal mucosal cell apoptosis. Cell Death Dis. (2017) 8:e3127–7. doi: 10.1038/cddis.2017.508, PMID: 29022896 PMC5682673

[14] Zhao X . Apigenin-7-glucoside-loaded nanoparticle alleviates intestinal ischemia-reperfusion by ATF3/SLC7A11-mediated ferroptosis. J Controlled Release. (2024). doi: 10.1016/j.jconrel.2023.12.038, PMID: 38145659

[15] Lu L-Q Li M-R Liu X-Y Peng D Liu H-R Zhang X-J . CARD11-BCL10-MALT1 Complex-Dependent MALT1 Activation Facilitates Myocardial Oxidative Stress in Doxorubicin-Treated Mice via Enhancing k48-Linked Ubiquitination of Nrf2. Antioxidants Redox Signaling. (2024), ars.2023.0543. doi: 10.1089/ars.2023.0543, PMID: 38814831

[16] Chiu CJ McArdle AH Brown R Scott HJ Gurd FN . Intestinal mucosal lesion in low-flow states. I. A morphological, hemodynamic, and metabolic reappraisal. Arch Surg. (1970) 101:478–83. doi: 10.1001/archsurg.1970.01340280030009, PMID: 5457245

[17] Ge Y Zadeh M Mohamadzadeh M . Vitamin B12 coordinates ileal epithelial cell and microbiota functions to resist *Salmonella* infection in mice. J Exp Med. (2022) 219:e20220057. doi: 10.1084/jem.20220057, PMID: 35674742 PMC9184849

[18] Iida T Takagi T Katada K Mizushima K Fukuda W Kamada K . Rapamycin improves mortality following intestinal ischemia-reperfusion via the inhibition of remote lung inflammation in mice. Digestion. (2015) 92:211–9. doi: 10.1159/000439300, PMID: 26402062

[19] Lebek S Chemello F Caravia XM Tan W Li H Chen K . Ablation of CaMKIId oxidation by CRISPR-Cas9 base editing as a therapy for cardiac disease. Science. (2023) 379:179–85. doi: 10.1126/science.ade1105, PMID: 36634166 PMC10150399

[20] Wang D Yu X Gao K Li F Li X Pu H . Sweroside alleviates pressure overload-induced heart failure through targeting CaMKIIδ to inhibit ROS-mediated NF-κB/NLRP3 in cardiomyocytes. Redox Biol. (2024) 74:103223. doi: 10.1016/j.redox.2024.103223, PMID: 38851078 PMC11219961

[21] Ding R Wu W Sun Z Li Z . AMP-activated protein kinase: An attractive therapeutic target for ischemia-reperfusion injury. Eur J Pharmacol. (2020) 888:173484. doi: 10.1016/j.ejphar.2020.173484, PMID: 32798506

[22] Mao S Liu Z Tian Y Li D Gao X Wen Y . Branched-long-chain monomethyl fatty acids: are they hidden gems? J Agric Food Chem. 71:18674–84. doi: 10.1021/acs.jafc.3c06300, PMID: 37982580 PMC10705331

[23] Zhang Q . Dexmedetomidine inhibits mitochondria damage and apoptosis of enteric glial cells in experimental intestinal ischemia/reperfusion injury via SIRT3-dependent PINK1/HDAC3/p53 pathway. J Transl Med. (2021) 19:463. doi: 10.1186/s12967-021-03027-6, PMID: 34772407 PMC8588684

[24] Liao S Luo J Kadier T Ding K Chen R Meng Q . Mitochondrial DNA release contributes to intestinal ischemia/reperfusion injury. Front Pharmacol. (2022) 13:854994. doi: 10.3389/fphar.2022.854994, PMID: 35370747 PMC8966724

[25] Lv S Zhao X Ma C Zhao D Sun T Fu W . Advancements in the study of acute lung injury resulting from intestinal ischemia/reperfusion. Front Med. 11:1399744. doi: 10.3389/fmed.2024.1399744, PMID: 38933104 PMC11199783

[26] Stoyanova I Lutz D . Ghrelin-mediated regeneration and plasticity after nervous system injury. Front Cell Dev Biol. (2021) 9:595914. doi: 10.3389/fcell.2021.595914, PMID: 33869167 PMC8046019

[27] Su B-C Li C-C Horng J-L Chen J-Y . Calcium-dependent calpain activation-mediated mitochondrial dysfunction and oxidative stress are required for cytotoxicity of epinecidin-1 in human synovial sarcoma SW982 cells. IJMS. (2020) 21:2109. doi: 10.3390/ijms21062109, PMID: 32204400 PMC7139453

[28] Bauer TM Murphy E . Role of mitochondrial calcium and the permeability transition pore in regulating cell death. Circ Res. (2020) 126:280–93. doi: 10.1161/CIRCRESAHA.119.316306, PMID: 31944918 PMC8317591

[29] Kiwit A Lu Y Lenz M Knopf J Mohr C Ledermann Y . The dual role of neutrophil extracellular traps (NETs) in sepsis and ischemia-reperfusion injury: comparative analysis across murine models. Int J Mol Sci. (2024) 25:3787. doi: 10.3390/ijms25073787, PMID: 38612596 PMC11011604

[30] Huang C Gurlo T Haataja L Costes S Daval M Ryazantsev S . Calcium-activated calpain-2 is a mediator of beta cell dysfunction and apoptosis in type 2 diabetes. J Biol Chem. (2010) 285:339–48. doi: 10.1074/jbc.M109.024190, PMID: 19861418 PMC2804181

[31] Wang B Dudko OK . A theory of synaptic transmission. eLife. (2021) 10:e73585. doi: 10.7554/eLife.73585, PMID: 34970965 PMC8776255

[32] Zhang H Lei M Zhang Y Li H He Z Xie S . Phosphorylation of Doc2 by EphB2 modulates Munc13-mediated SNARE complex assembly and neurotransmitter release. Sci Adv. (2024) 10:eadi7024. doi: 10.1126/sciadv.adi7024, PMID: 38758791 PMC11100570

[33] DalleDonne I Milzani A Colombo R . Actin assembly by cadmium ions. Biochim Biophys Acta (BBA) - Mol Cell Res. (1997) 1357:5–17. doi: 10.1016/S0167-4889(97)00008-6, PMID: 9202170

[34] Princen K Dooren TV van Gorsel M Louros N Yang X Dumbacher M . Pharmacological modulation of septins restores calcium homeostasis and is neuroprotective in models of Alzheimer’s disease. Science. (2024) 384:eadd6260. doi: 10.1126/science.add6260, PMID: 38815015 PMC11827694

[35] Sampedro-Castañeda M . Epilepsy-linked kinase CDKL5 phosphorylates voltage-gated calcium channel Cav2.3, altering inactivation kinetics and neuronal excitability. Nat Commun. (2023) 14:7830. doi: 10.1038/s41467-023-43475-w, PMID: 38081835 PMC10713615

[36] Lebek S Caravia XM Chemello F Tan W McAnally JR Chen K . Elimination of caMKIIδ Autophosphorylation by CRISPR-cas9 base editing improves survival and cardiac function in heart failure in mice. Circulation. (2023) 148:1490–504. doi: 10.1161/CIRCULATIONAHA.123.065117, PMID: 37712250 PMC10842988

[37] Lebek S Caravia XM Straub LG Alzhanov D Tan W Li H . CRISPR-Cas9 base editing of pathogenic CaMKIIδ improves cardiac function in a humanized mouse model. J Clin Invest. (2024) 134:e175164. doi: 10.1172/JCI175164, PMID: 37856214 PMC10760954

[38] Xu X Yang D Ding J-H Wang W Chu P-H Dalton ND . ASF/SF2-regulated caMKIIδ Alternative splicing temporally reprograms excitation-contraction coupling in cardiac muscle. Cell. (2005) 120:59–72. doi: 10.1016/j.cell.2004.11.036, PMID: 15652482

[39] Cunningham KE Novak EA Vincent G Siow VS Griffith BD Ranganathan S . Calcium/calmodulin–dependent protein kinase IV (CaMKIV) activation contributes to the pathogenesis of experimental colitis *via* inhibition of intestinal epithelial cell proliferation. FASEB J. (2019) 33:1330–46. doi: 10.1096/fj.201800535R, PMID: 30113881 PMC6355073

[40] Shiomi R Shigetomi K Inai T Sakai M Ikenouchi J . CaMKII regulates the strength of the epithelial barrier. Sci Rep. (2015) 5:13262. doi: 10.1038/srep13262, PMID: 26281891 PMC4539604

[41] Bao X Gänzle MG Wu J . Ovomucin hydrolysates reduce bacterial adhesion and inflammation in enterotoxigenic *escherichia coli* (ETEC) K88-challenged intestinal epithelial cells. J Agric Food Chem. (2024) 72:7219–29. doi: 10.1021/acs.jafc.4c00185, PMID: 38507577

[42] Guo N-K She H Tan L Zhou Y-Q Tang C-Q Peng X-Y . Nano parthenolide improves intestinal barrier function of sepsis by inhibiting apoptosis and ROS via 5-HTR2A. IJN. (2023) 18:693–709. doi: 10.2147/IJN.S394544, PMID: 36816330 PMC9930579

[43] Gou H Su H Liu D Wong CC Shang H Fang Y . Traditional medicine pien tze huang suppresses colorectal tumorigenesis through restoring gut microbiota and metabolites. Gastroenterology. (2023) 165:1404–19. doi: 10.1053/j.gastro.2023.08.052, PMID: 37704113

[44] Zhang T Zhang Y Cui M Jin L Wang Y Lv F . CaMKII is a RIP3 substrate mediating ischemia- and oxidative stress–induced myocardial necroptosis. Nat Med. (2016) 22:175–82. doi: 10.1038/nm.4017, PMID: 26726877

[45] Chen W An P Quan X-J Zhang J Zhou Z-Y Zou L-P . Ca^2+^ /calmodulin-dependent protein kinase II regulates colon cancer proliferation and migration *via* ERK1/2 and p38 pathways. WJG. (2017) 23:6111–8. doi: 10.3748/wjg.v23.i33.6111, PMID: 28970726 PMC5597502

[46] Salas MA Valverde CA Sánchez G Said M Rodriguez JS Portiansky EL . The signaling pathway of CaMKII-mediated apoptosis and necrosis in the ischemia/reperfusion injury. J Mol Cell Cardiol. (2010) 48:1298–306. doi: 10.1016/j.yjmcc.2009.12.015, PMID: 20060004 PMC2866824

[47] Wu J Xu X Duan J Chai Y Song J Gong D . EFHD2 suppresses intestinal inflammation by blocking intestinal epithelial cell TNFR1 internalization and cell death. Nat Commun. (2024) 15:1282. doi: 10.1038/s41467-024-45539-x, PMID: 38346956 PMC10861516

[48] Parker A Vaux L Patterson AM Modasia A Muraro D Fletcher AG . Elevated apoptosis impairs epithelial cell turnover and shortens villi in TNF-driven intestinal inflammation. Cell Death Dis. (2019) 10:108. doi: 10.1038/s41419-018-1275-5, PMID: 30728350 PMC6365534

[49] Liu M Rao H Liu J Li X Feng W Gui L . The histone methyltransferase SETD2 modulates oxidative stress to attenuate experimental colitis. Redox Biol. (2021) 43:102004. doi: 10.1016/j.redox.2021.102004, PMID: 34020310 PMC8141928

[50] Hu J Shi D Ding M Huang T Gu R Xiao J . Calmodulin-dependent signaling pathways are activated and mediate the acute inflammatory response of injured skeletal muscle. J Physiol. (2019) 597:5161–77. doi: 10.1113/JP278478, PMID: 31506936

[51] Guo X Xu J Huang C Zhang Y Zhao H Zhu M . Rapamycin extenuates experimental colitis by modulating the gut microbiota. J Gastroenterol Hepatol. (2023) 38:2130–41. doi: 10.1111/jgh.16381, PMID: 37916431

[52] Boada C Zinger A Tsao C Zhao P Martinez JO Hartman K . Rapamycin-loaded biomimetic nanoparticles reverse vascular inflammation. Circ Res. (2020) 126:25–37. doi: 10.1161/circresaha.119.315185, PMID: 31647755

