# Supplementary figures and images for: Bioinformatics-driven insights: rapamycin-mediated CaMK2D inhibition alleviates intestinal ischemia-reperfusion injury
Source: Front Immunol. 2026 May 1;16:1684853. doi: 10.3389/fimmu.2025.1684853 (PMC13175813; doi:10.3389/fimmu.2025.1684853)

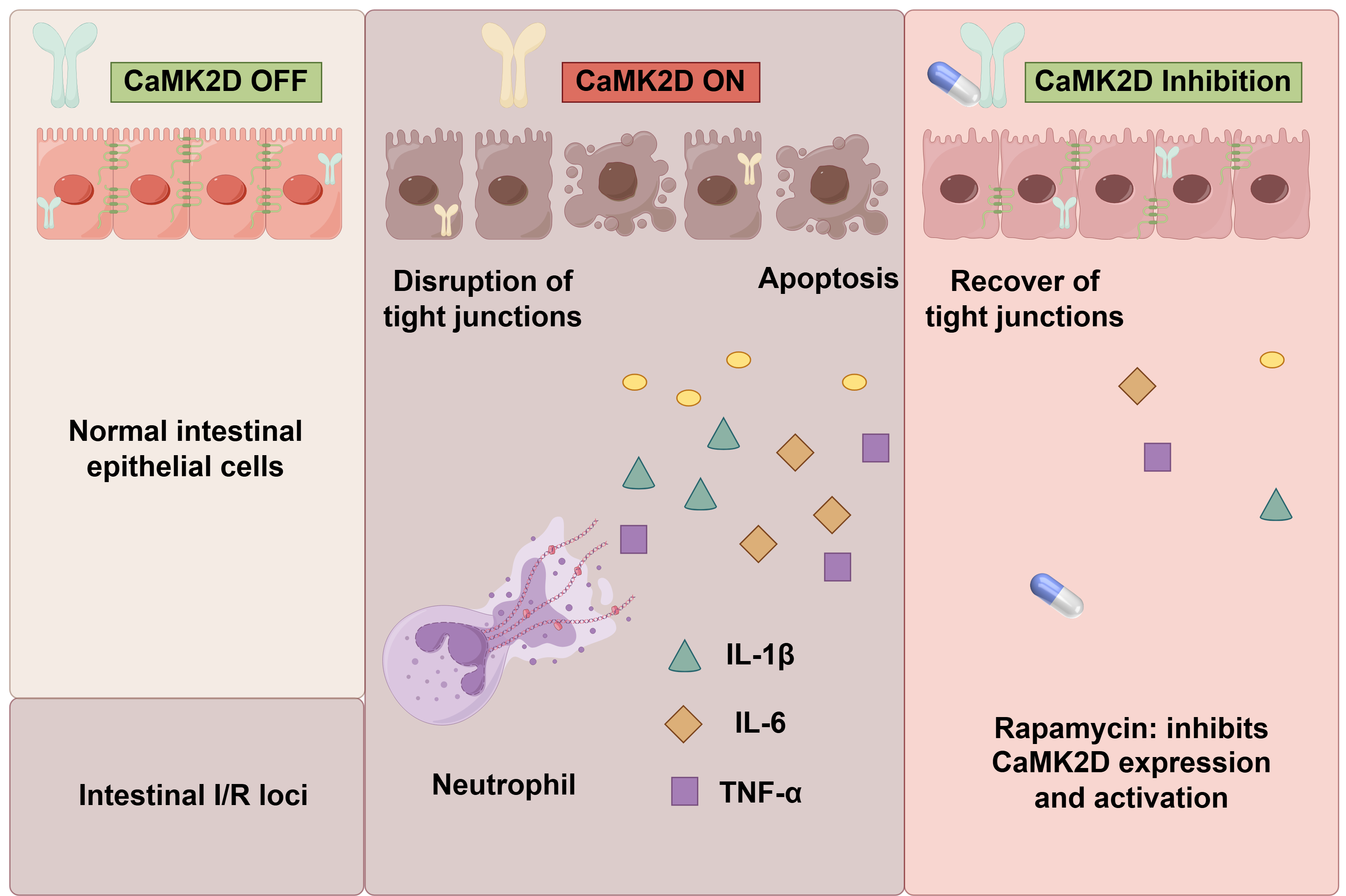

Supplement: Supplementary file 1 [file Image1.png]
