# Supplementary material for: Bioinformatics-driven insights: rapamycin-mediated CaMK2D inhibition alleviates intestinal ischemia-reperfusion injury
Source: Front Immunol. 2026 May 1;16:1684853. doi: 10.3389/fimmu.2025.1684853 (PMC13175813; doi:10.3389/fimmu.2025.1684853)
Supplement: Supplementary file 3 [file Table1.docx]

**Supplementary information**

**Bioinformatics-Driven Insights: Rapamycin-Mediated CaMK2D inhibition Alleviates Intestinal Ischemia-Reperfusion Injury**

Ruxiang Sheng^1^**^†^**, Yanqiu Liang^1^**^†^**, Huihong Zhang^1^, Yonghe Lai^1^, Haiyu Hong^1*^, Dezhao Liu^1*^

^1^ Department of Anesthesiology, Fifth Affiliated Hospital of Sun Yat-Sen University

**^†^**These authors contributes equally.

Associate authors: [honghy@sysu.edu.cn](mailto:honghy@sysu.edu.cn,), Haiyu Hong; [sumsldz@126.com](mailto:sumsldz@126.com), Dezhao Liu


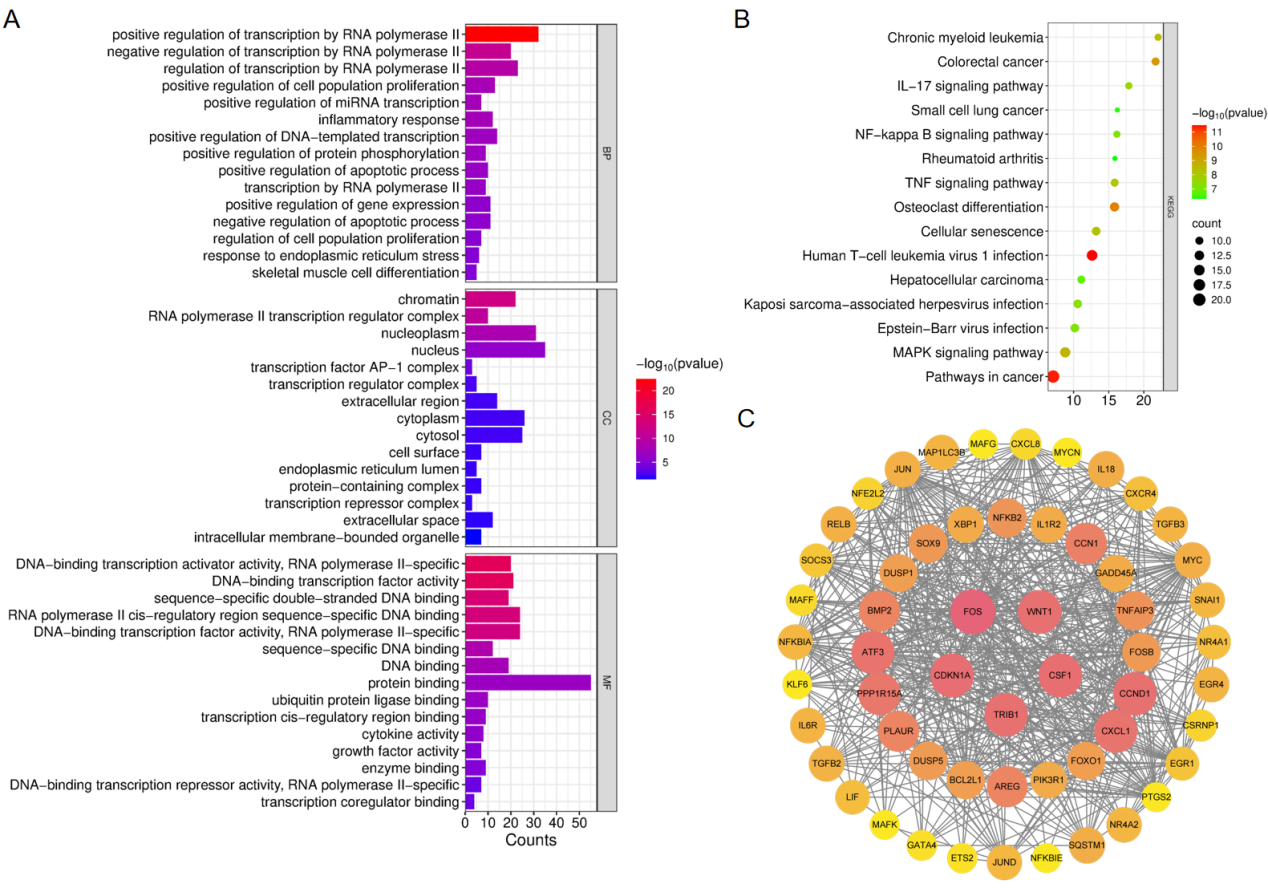


**Supplementary Figure 1.** GO functional enrichment, KEGG pathway, and PPI network analysis of Cluster 1. (A) GO functional enrichment analysis of Cluster 1. (B) KEGG pathway enrichment analysis of Cluster 1. (C) Protein-protein interaction network of Cluster 1.


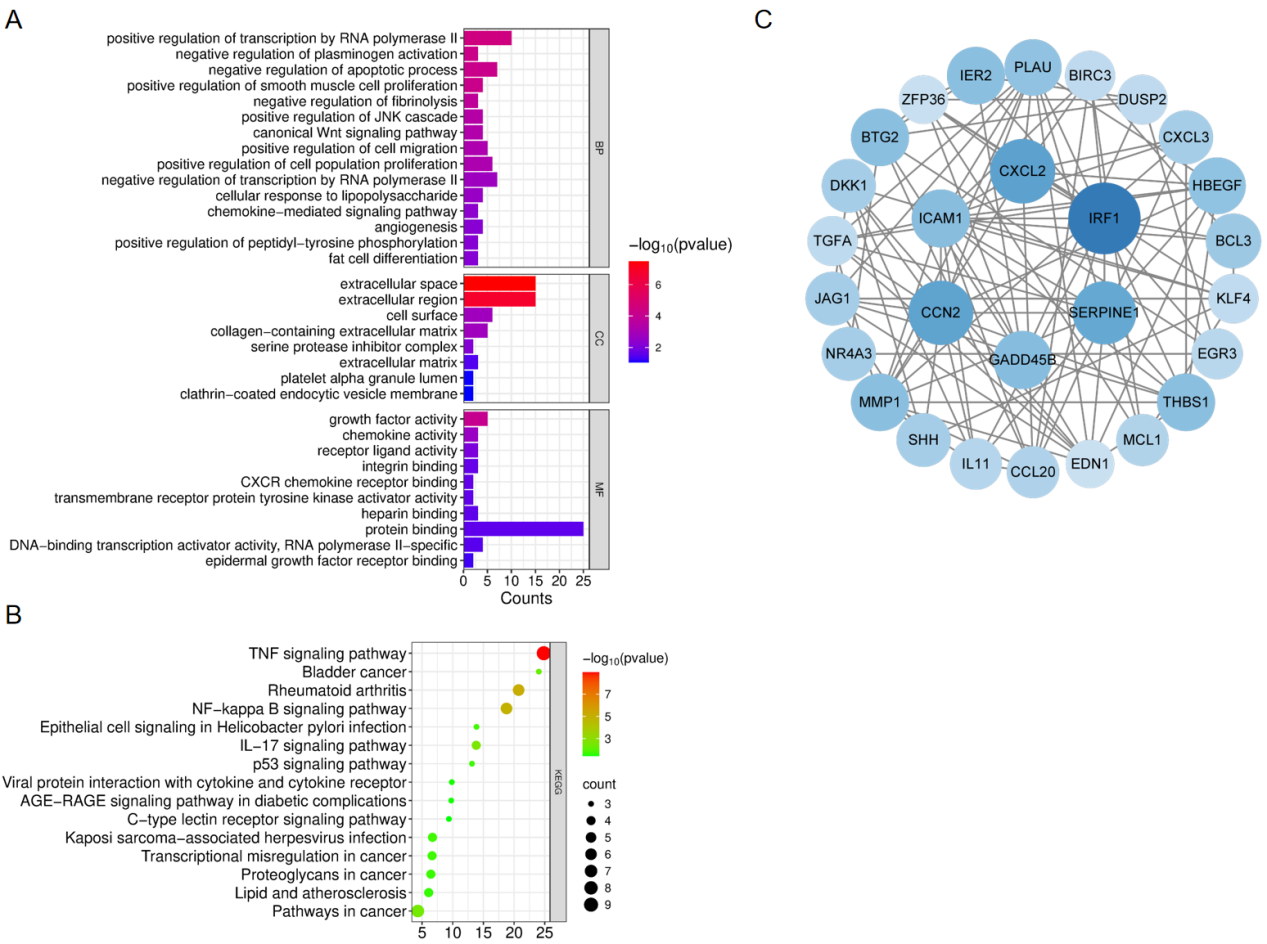


**Supplementary Figure 2.** GO functional enrichment, KEGG pathway, and PPI network analysis of Cluster 2. (A) GO functional enrichment analysis of Cluster 2. (B) KEGG pathway enrichment analysis of Cluster 2. (C) Protein-protein interaction network of Cluster 2.


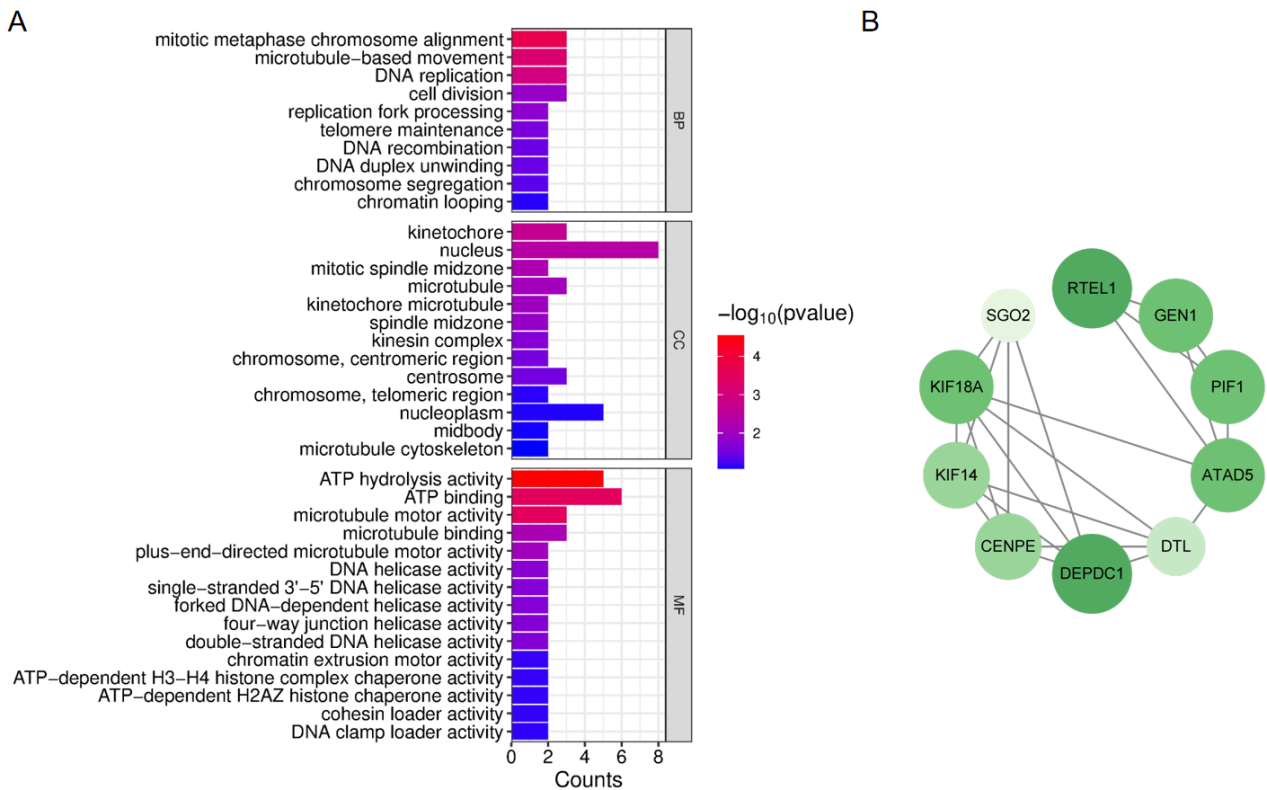


**Supplementary Figure 3.** GO functional enrichment, KEGG pathway, and PPI network analysis of Cluster 3. (A) GO functional enrichment analysis of Cluster 3. (B) Protein-protein interaction network of Cluster 3.


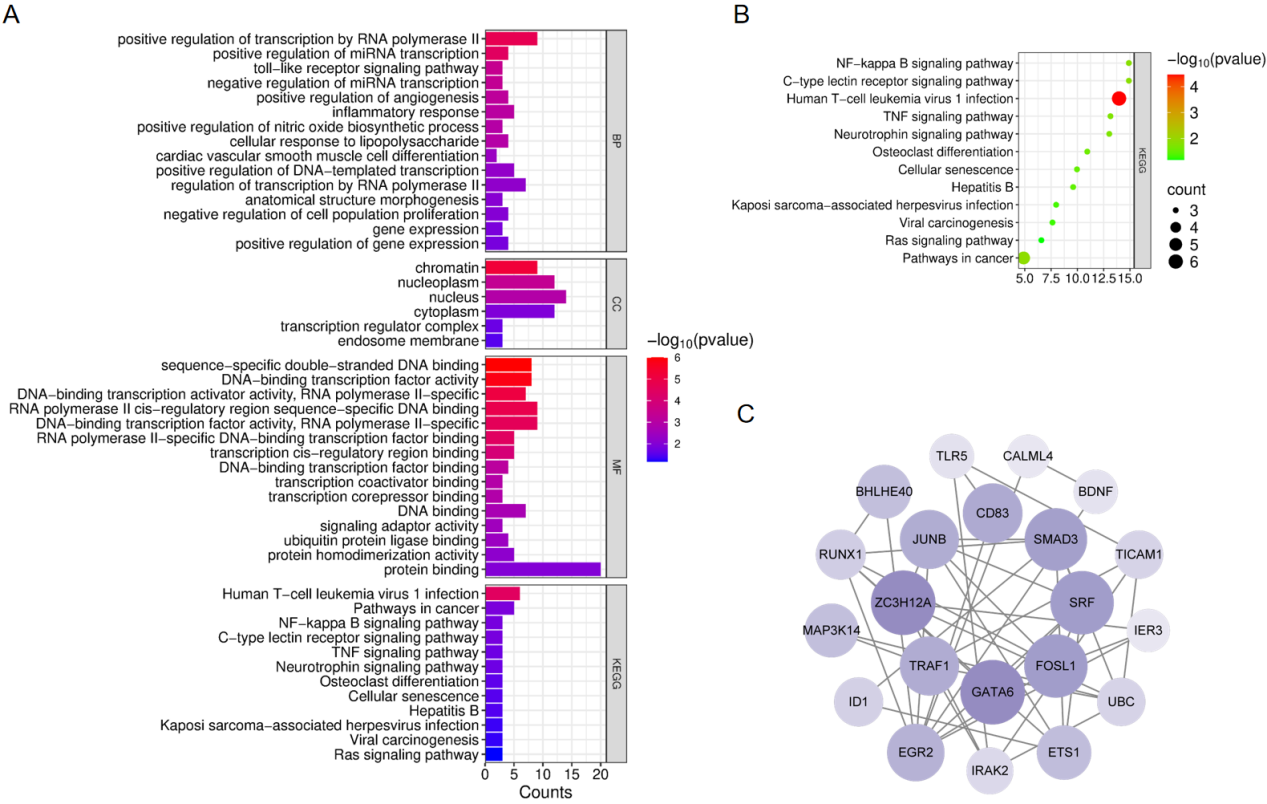


**Supplementary Figure 4.** GO functional enrichment, KEGG pathway, and PPI network analysis of Cluster 4. (A) GO functional enrichment analysis of Cluster 4. (B) KEGG pathway enrichment analysis of Cluster 4. (C) Protein-protein interaction network of Cluster 4.


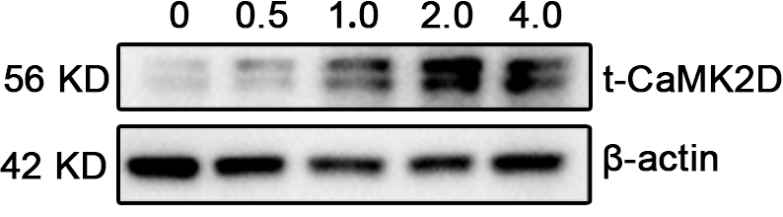


**Supplementary Figure 5.** Dose-dependent expression of t-CaMK2D after transfection with overexpression plasmid. Caco-2 cells were transfected with CaMK2D overexpression plasmid at 0, 0.5, 1.0, 2.0, 4.0 μg/well (6-well plate), and protein levels were detected by Western blot with β-actin as internal control (n=3).
